# Combined sensory, volatilome and transcriptome analyses identify a limonene terpene synthase as a major contributor to the characteristic aroma of a *Coffea arabica* L. specialty coffee

**DOI:** 10.1186/s12870-024-04890-3

**Published:** 2024-04-03

**Authors:** Lison Marie, Jean-Christophe Breitler, Pingdwende Kader Aziz Bamogo, Mélanie Bordeaux, Séverine Lacombe, Maëlle Rios, Marc Lebrun, Renaud Boulanger, Eveline Lefort, Sunao Nakamura, Yudai Motoyoshi, Delphine Mieulet, Claudine Campa, Laurent Legendre, Benoît Bertrand

**Affiliations:** 1 CIRAD (Centre de coopération internationale en recherche agronomique pour le développement), UMR DIADE, Montpellier, F-34398 France; 2 DIADE (Diversity, Adaptation, Development of Plants), University of Montpellier, CIRAD, IRD, Montpellier, F-34398 France; 3 PHIM (Plant Health Institute of Montpellier), University of Montpellier, CIRAD, IRD, INRAE, Institut Agro, Montpellier, F-34398 France; 4 FUNDACION NICAFRANCE, Matagalpa, Nicaragua; 5 CIRAD, UMR QualiSud, Montpellier, F-34398 France; 6 QualiSud, University of Montpellier, CIRAD, IRD, INRAE, Institut Agro, University of La Réunion, University of Avignon, Montpellier, F-34398 France; 7 Research Institute, Suntory Global Innovation Center Limited, 8-1-1, Seika-dai, Seika-cho, Soraku-gun Kyoto, 619-0284 Japan; 8 INRAE, UR 1115 Plantes et Systèmes de Culture Horticoles, Site Agroparc, Avignon, 84914 France

**Keywords:** *Coffea arabica*, Coffee quality, Flavors, Genotypes, Metabolomics, Monoterpenes, Terpene synthases

## Abstract

**Background:**

The fruity aromatic bouquet of coffee has attracted recent interest to differentiate high value market produce as specialty coffee. Although the volatile compounds present in green and roasted coffee beans have been extensively described, no study has yet linked varietal molecular differences to the greater abundance of specific substances and support the aroma specificity of specialty coffees.

**Results:**

This study compared four Arabica genotypes including one, Geisha Especial, suggested to generate specialty coffee. Formal sensory evaluations of coffee beverages stressed the importance of coffee genotype in aroma perception and that Geisha Especial-made coffee stood out by having fine fruity, and floral, aromas and a more balanced acidity. Comparative SPME–GC–MS analyses of green and roasted bean volatile compounds indicated that those of Geisha Especial differed by having greater amounts of limonene and 3-methylbutanoic acid in agreement with the coffee cup aroma perception. A search for gene ontology differences of ripening beans transcriptomes of the four varieties revealed that they differed by metabolic processes linked to terpene biosynthesis due to the greater gene expression of prenyl-pyrophosphate biosynthetic genes and terpene synthases. Only one terpene synthase (*Ca*TPS10-like) had an expression pattern that paralleled limonene loss during the final stage of berry ripening and limonene content in the studied four varieties beans. Its functional expression in tobacco leaves confirmed its functioning as a limonene synthase.

**Conclusions:**

Taken together, these data indicate that coffee variety genotypic specificities may influence ripe berry chemotype and final coffee aroma unicity. For the specialty coffee variety Geisha Especial, greater expression of terpene biosynthetic genes including *Ca*TPS10-like, a limonene synthase, resulted in the greater abundance of limonene in green beans, roasted beans and a unique citrus note of the coffee drink.

**Supplementary Information:**

The online version contains supplementary material available at 10.1186/s12870-024-04890-3.

## Background

Coffee is the most popular stimulating beverage worldwide. With a total consumption of ~ 170 million 60 kg bags in 2021/22 [1], it surpassed chocolate and tea commodities and has grown most rapidly since 2000 with new emerging markets in China and South Korea [2]. Most production takes place between the Tropics of Cancer and Capricorn, in a 'bean belt' of over 100 million farmers [3]. Mostly two species are cultivated, *Coffea arabica* L. (Arabica) and *C. canephora* Pierre ex a. Froehner (Robusta). Despite its greater sensitivity to biotic and abiotic stresses, Arabica accounted in 2021/22 for ~ 56% of the world production [1]. Tolerance to stress and productivity have historically been the main drivers of coffee genetic selection and improvement so that modern cultivars have poorer flavor [4]. Nevertheless, recent changes in consumer preferences are shifting interest towards the selection of 'specialty' coffee cultivars with improved aromatic quality.

Coffee beverage quality is a complex trait involving a blend of natural substances of different biosynthetic origins influencing scent, aroma and taste. The Arabica fruit takes approximately 240 days after anthesis to ripen [5]. Coffee beans consist largely of the endosperm, the reserve tissue, which makes up more than 98% of the dry matter of mature seeds [6]. The storage components are mainly polysaccharides, lipids, proteins and sucrose. The bean also contains significant amounts of specialized metabolites, including alkaloids (caffeine, trigonelline) and phenolics (chlorogenic acids) respectively responsible for its bitterness and stringency [7–9]. Fermentation of the fresh coffee beans modifies their chemical composition, allowing the fermented beans, known as the green bean, to contain the precursors of the Maillard and Strecker reactions taking place during roasting and that give a final modification of the aroma [10]. Green coffee beans contain c.a. 300 volatile compounds belonging to different classes (notably phenolics, terpenes, amino acid derivatives). The number of identified aromatic volatile scent substances rises to 1000 after roasting [11, 12], including some already present in the green beans and therefore conserved during roasting [13]*. *Surprisingly few studies have analyzed the nature of the aromatic substances present in the final coffee beverage [14–16]*. *All result from a transfer from the roasted beans to the hot beverage. Knowing the relationship between harvested and roasted bean components, it is not surprising that many factors ranging from tree genetics, post-harvest treatments and brewing conditions have been found to influence coffee beverage aroma substances and coffee beverage perception [17, 18]. Unlike in other cultivated plant species, the molecular determinants of coffee aromatic scent have not yet received substantial interest [19], complicating the work of breeders in their quest for marker to assist selection steps.

Recent technological and bioinformatics advances have allowed unprecedented progresses in our knowledge of the chemical composition (metabolomics) and level of gene expression (transcriptomics) of plants [20]. It is a powerful approach for identifying the genetic determinants of a chemical phenotype of interest. Numerous studies have highlighted key genes using these methods. Terpene synthesis genes involved in the synthesis of linalool, which contributes to the typical aroma of muscat-type grapes, have been highlighted [21]. Chen et al. 2020 showed the regulatory effects of methyl jasmonate on flavonoid biosynthesis genes leading to the accumulation of quinone chalcone in safflower [22]. In addition, transcription factors controlling the formation of volatile esters during pear ripening [23] and carotenoid biosynthesis genes involved in the formation of mango aroma during ripening [24] have been identified. It has also been shown that odor emission in the fragrant *Lonicera japonica* is under the control of terpene biosynthesis genes [25].

In order to unveil the *Coffea* genomic determinants of the improved sensory evaluation of coffee beverages made from the specialty genotype Geisha Especial, the precise sensory characteristics of such beverages were first formally established by specialized taste panels. Coffee cups made from this genotype were compared to those of three other genotypes know to be of lesser appraisal. All genotypes were grown under similar conditions to prevent environmental effects [26–31]. A comparative analysis of the volatile organic compounds of the green and roasted beans of these four genotypes was then conduced to associate coffee cup taste perception to the greater content of a specific, or specific bouquet of, aroma substance(s). Similarly, comparative transcriptomics combined to gene ontology search was conducted to link volatile substance emission to the differential expression of volatile substance biosynthetic pathways. The functional analysis of the candidate genes involved in the key step of carbon skeleton formation was finally carried out to support previous conclusions.

## Materials and methods

### Plant materials and samples processing

Four Arabica pure line genotypes were studied. These were two Ethiopians (Geisha Especial and ET47) and two *C. arabica* introgressed *C. canephora* (T5175, a Catimor – Timor hybrids x Caturra – and Marsellesa, a Sarchimor – Timor hybrids x Villa Sarchi –). Geisha Especial is an Ethiopian landrace introduced in East Africa (Kenya, Tanzania) from Ethiopia and then to Costa Rica (CATIE collection) and then to Panama. The Geisha cultivar we used was selected by Dr C. M. Rodriguez, from the Starbucks Company, for its outstanding aroma and flavor. For this reason, it was named ‘Geisha Especial’. Seeds of the cultivar Geisha Especial were provided by the farm La Alsacia, Costa Rica. M. Bordeaux, from The NicaFrance foundation (Nicaragua), provided seeds of the three others cultivars. ET47 is a wild accession, indigenous to Ethiopia, identified during the Orstom survey in 1968. B. Bertrand as part of the Promecafe project introduced this accession to Nicaragua in 1992. This accession exhibits a complex flavor profiles as many other Ethiopian accessions or landraces [32]. Geisha Especial and ET47 are Arabica Ethiopian accession, indigenous to Ethiopia. T5175 is the Turrialba T accession from the CATIE collection (Costa Rica), introduced into this collection by Promecafe. T5175 was brought to Nicaragua by Promecafe in 1982. Promecafe is an institution under the auspices of the Inter-American Institute for Agricultural Cooperation (IICA). T5175 also known as IHCAFE90 has an aromatic profile that can be described as very poor. B. Bertrand and C. Ponçon, from CIRAD and ECOM respectively, selected Marsellesa in Nicaragua. This variety was added to the UPOV catalogue in 2010. Marsellesa is a cultivar that develops an aromatic profile considered as good, though not exceptional. T5175 and Marsellesa are pure lines deriving from crosses between Timor hybrid (a natural interspecific hybrid between *C. arabica* and *C. canephora*). Crosses between Timor hybrids and Arabica allowed the process of *C. canephora* genes introgression into Arabica cultivars. Crosses between Timor hybrids and traditional cultivars such as Caturra or Villa Sarchi followed by a backcross with the same cultivar and then pedigree selection, gave rise to respectively, Catimor (i.e. T5175) and Sarchimor (i.e. Marsellesa). All four cultivars were cultivated at 1300 m on La Cumplida farm in Nicaragua. Three trees of each genotype were planted in barrels and randomly placed under a transparent shelter to control watering, fertilization, flowering initiation, and to reduce environmental variability (Fig. S1).

For the transcriptomic analyses, coffee fruits harvested on a similar tree, cultivar and ripening stage constituted a sample (total of 24 samples = 3 trees × 4 genotypes × 2 ripening stages). The fruits were pulped upon harvest and the beans immediately dipped in liquid nitrogen.

For the biochemical and sensory analyses, approximately 1 kg of coffee fruit samples were harvested per tree for each of the four genotypes (except with Geisha Especial for which a tree had died a few months before harvest) at full maturity (total of 11 samples = 2–3 trees × 4 genotypes × 1 ripening stage). Freshly harvested beans were transported directly to the processing mill and processed with the wet method (pulping, overnight fermentation, washing and drying). Once the beans were dried at 11% humidity, they went through a hulling process, and were then sorted and graded based on size and quality. Defective beans were eliminated to obtain the final samples of green coffee beans (150 g of green coffee beans per tree). Subsamples of 15 g were subjected to volatile compounds analysis while the remaining 100 g were taken for roasting. After roasting, the resulting 80 g of roasted beans were divided as follows: 15 g for volatile compounds analysis and 65 g for the sensory analysis. All samples were roasted simultaneously in order to minimize roasting process-associated variability and left to stand at ambient temperature for a minimum of 12 h to allow for degassing (mostly CO2). The samples for sensory evaluation were vacuum-sealed before dispatch.

### Sensory analysis

The three 65 g samples of roasted beans originating from the three trees of a similar cultivar were mixed to yield 195 g of roasted beans. The four genotype samples were evaluated by two independent panels (Nespresso, Switzerland and Jacobs Douwe Egberts (JDE), Netherlands), each consisting of four judges. Each of the two panels received 90 g of roasted beans. The samples were ground to a medium/fine powder immediately prior to cupping. For each sample, 15 g of ground coffee was confronted to 300 mL of boiled water (~ 95°C). A Bodum French press coffee maker (i.e., an immersion brewing method) was used to prepare the beverage. The hot water was poured directly onto the measured ground coffee, making sure to wet the grounds thoroughly. Shortly afterwards, once the coffee solids had risen to the surface of the French press, the mixture was stirred to homogenize the coffee slurry. The mixture was left to stand for exactly 5 min before pouring 60 mL aliquots of the supernatant into cups. Sensory analysis was undertaken when the beverage temperature had reached 50 to 55°C. Each sample was assessed blindly and evaluated by each of the 2 × 4 judges. Each judge had his/her own set of samples. Evaluation was conducted by aspirating the coffee into the mouth, directly from the sampling cup, to take the vapour and liquid to the tongue and upper palate. The samples were evaluated using a protocol developed by CIRAD with modifications from the European standards ISO 6668 and 13299 (hereafter referred to as the CIRAD sensory protocol) (Table S1). The following attributes were scored (each out of 10 points) by the judges: acidity and fruity (positive attributes), greeny (medium attribute), harsh and bitterness (negative attributes). In addition, members of both panels were invited to make open comments.

### Volatile compounds analysis of green and roasted beans by SPME–GC–MS

#### Extraction of volatile compounds by headspace-SPME

Samples of green beans and roasted beans were ground to a fine powder separately for each tree (biological replicates). Green coffee beans were ground with dry ice in an IKA Grinder Tube Mill (IKA, Staufen, Germany). Roasted coffee beans were ground in an Ultra Centrifugal Mill ZM 200 (Retsch-ZM200, France). Two grams of ground coffee were placed in a hermetically sealed 10 mL glass vial containing 1-butanol (100 µL/2 g) as internal standard (Sigma-Aldrich) (5 µL/100 mL). Three vials of 2 g were prepared per biological replicate. A DVB/CAR/PDMS (Divinylbenzene/Carboxen/Polydimethylsiloxane, 50/30 µm Stableflex fibre) SPME fiber (Supelco Co., Bellefonte, PA, USA) was introduced in the glass vials that had been previously placed in a thermostatically regulated oven for 15 min at 50°C to reach sample headspace equilibrium. The sample headspaces were then extracted with SPME for 45 min at 50°C. Three independent extractions (technical replicates) were made from each powder sample corresponding to one tree (biological replicate).

#### Combined gas chromatography–mass spectroscopy

The volatile compounds were analysed with an Agilent 6890 gas chromatograph coupled to an Agilent 5973 mass spectrometer (Agilent Technologies, Palo Alto, USA). SPME fibres were desorbed (Desorption Time: 120 s) in splitless mode with the injector temperature set at 250 °C. Desorbed substances were then separated in a DB-WAX polar column (60 m × 0.25 mm, 0.25 μm phase film thickness, Agilent J&W GC column, USA). Hydrogen was used as the carrier gas with a constant flow rate of 1.5 mL/min. The initial oven temperature was 40°C (5 min) followed by a 2°C/min increase up to 170°C and a 10°C/min increase to 250°C where temperature was kept constant for 10 min. The mass spectrometer operated at 70 eV in electron impact (EI) ionization mode. The ionization source was heated at 230°C. After ionization, the molecules were separated according to their mass/charge (m/z) by a quadrupole analyser maintained at 150°C and programmed to regularly scan (4.51 scans/sec) a mass range of [40 to 350] m/z. The set of results was processed using MassHunter Qualitative Analyses software and MS Quantitative Analysis software (version 10.2.1).

#### Volatile compounds identification and quantitation

Identification of volatile compounds was based on similarities of mass spectra (%) with those present in the National Institute of Standards and Technology (NIST, Gaithersburg, Maryland, United States) database and by comparison of their calculated retention indices to those available in NIST library (Tables S2 and S3). The identification of some compounds was confirmed by the parallel use of authentic standards. The chirality of enantiomeric substances was not determined so that substances such as limonene are either ( +)-limonene or (-)-limonene.

Semi-quantitative estimates of substance contents (mi) were based on peak areas expressed as percentages of the one of 1-butanol to normalize the different extracts using the following formula: mi (μg/g of dry mass) = Ki/KEI x Ai/AEI x mEI/mp × 100, where Ki is the coefficient of response of the unknown molecule, KEI is the coefficient of response of the internal standard, Ai is the peak area of the volatile compound, AEI is the peak area of butanol, mEI is the quantity of butanol, mp is the quantity of green or roasted powder. Calculations were performed by assuming Ki/KEI = 1, as previously described by Flowers et al. (2022) [33]. For each volatile substance, the mean of the three technical replicates values was used for downstream statistical analyses.

### RNA library preparation, sequencing and RNA-Seq transcriptome analysis

#### RNA extraction

Fruits were sampled at the last two ripening stages, during maturation of the pericarp, around 210 days after flowering (DAF) (hereafter ‘yellow stage’) (stage 6) and around 240 DAF (hereafter ‘red stage’) (stage 7). Each of the three biological replicates contained beans from a different tree. Twenty beans were sampled from different sides of the canopy of each tree. Representative portions of the fruit endosperm tissues were frozen in liquid nitrogen and stored at − 80°C until use. Total RNA was extracted by grinding 100 mg aliquots of frozen tissues to a fine powder in liquid nitrogen using the Qiagen "RNeasy® Lipid Tissue" kit (QIAGEN, Germantown, MD, USA). The quality and quantity of total RNA were estimated using an Agilent 2100 Bioanalyzer RNA chip (Agilent Technologies Inc., Santa Clara, CA—USA). The RNA samples with RNA integrity number (RIN) higher than 7.0 were selected and used for the subsequent analysis.

#### Illumina sequencing

RNA sequencing was performed by MGX (Montpellier, France). The cDNA libraries were generated using the TruSeq Stranded mRNA Kit (Illumina) followed by PCR amplification for sequencing on Illumina NovaSeq 6000. Paired-end cDNA libraries were generated from all samples and sequencing was performed to generate ~ 150 bp paired-end reads. Quality control and assessment of raw Illumina reads in FASTQ format were done by FastQC software (v0.11.8). Clean reads were obtained by removing low-quality reads, adapters, and poly-N-containing reads from the raw data. Approximately 95% of high quality reads were obtained from the raw generated data. On average, approximately 40 million paired-end reads were obtained for each library.

#### Read mapping and differential gene expression analysis

The pre-processed reads were aligned to the *Coffea arabica*: Cara 1.0 genome sequence (Johns Hopkins University) (NCBI RefSeq ID GCF_003713225.1) using TopHat v2.1.1 (with Bowtie 2.3.5.1)—paired-end 150. FeatureCounts (version 2.0.0) was used to count reads with positive match on genes. Differential expression analysis was used to determine how gene expression of target processes differed among conditions. For each transcript read, counts with a value ≥ 20 in at least 12% of the samples were considered as expressed and values were normalized using the R package EdgeR [34]. Sample libraries were normalized by calculating the effective library size and normalization factor using the TMM method on count data [35]. Subsequent counts were log2 transformed with a pseudo count of one and the resultant library was used for downstream analysis. A total of 26 746 genes were considered as expressed. Differentially expressed genes (DEGs) between pairwise comparisons (comparing stages by genotype, comparing genotypes by stage and cross comparison) in the bulk RNA-seq data were then identified using a generalized linear model (GLM) in the EdgeR package [34] with a cut-off of LogFC ≥ 1 for up-regulated genes and LogFC ≤ -1 for down-regulated genes, and FDR < 0.01.

#### Gene functional classification and enrichment analysis

Differentially expressed genes (DEGs) were compared against ‘The Arabidopsis Information Resource’ database (TAIR, www.arabidopsis.org) using BLASTP with the default settings. The resulting annotation was used to perform gene ontology (GO) analysis of DEGs. ClusterProfiler [36] and org.At.tair.db [37] packages were used to identify GO enriched terms amongst these genes. The GO enrichment analysis for Biological Processes was performed using the Bioconductor R library clusterProfiler, applying a hypergeometric test with FDR correction (adjusted *p* value < 0.05).

### Phylogenetic analyses

The amino acid sequences of terpene synthases from *Arabidopsis thaliana* (At), *Vitis vinifera* (Vv), *Solanum lycopersicum* (Sl) and *Coffea arabica* (Ca) were obtained from the National Center for Biotechnology Information database. Alignment of sequences was carried out with DECIPHER R package [38] and analysed by neighbor-joining (NJ) method (using the default settings) using the ape R package [39]. The phylogenetic tree was visualized using the ape R package [39].

### Agrobacterium-mediated transient expression of heterologous proteins in *Nicotiana benthamiana* leaves

#### Gene design, synthesis and cloning

The function of the terpene synthase 10-like (*Ca*TPS10-like) sequence from *C. arabica* L. cv. Caturra Red was determined by transient expression in *N. benthamiana* leaves. As positive controls, assays were conducted in parallel with *Ca*TPS1, a limonene synthase characterized in *C. arabica* L. cv. Catuai Red fruits [13], and *Sl*TPS7, a *beta*-Myrcene/Limonene synthase characterized by Zhou and Pichersky (2020) [40] in *Solanum lycopersicum* fruits. Genes DNA fragments were synthesized de novo by GenScript® (GenScript, HK Limited, Hong Kong) and cloned into the pBIN61 binary expression vector, under the control of the constitutive CaMV 35S promoter and terminator to generate pBIN61:*Ca*TPS10, pBIN61:*Ca*TPS1 and pBIN61:*Sl*TPS7 (Table S4).

#### Expression vectors and silencing suppressors

The experiments were performed using the *Agrobacterium tumefaciens* GV3101 (pMP90) strain harbouring the pBIN61 expression vector with sequences encoding constructs of interest or empty pBIN61 vectors as negative control. An additional pBIN61 expression vector carrying a gene encoding P19 from the *Cymbidium* ringspot virus (pBIN61:P19 vector) was used for the co-expression with pBIN61 vectors. P19 acts as a silencing suppressor and increases accumulation of proteins of interest in the *Agrobacterium*-mediated transient expression assays [41].

#### Agroinfiltration, plant material and experimental conditions

Strains harboring empty pBIN61, pBIN61:*Ca*TPS10, pBIN61:*Ca*TPS1, pBIN61:*Sl*TPS7 and pBIN61:P19 vectors were grown separately overnight at 28°C in an orbital shaker at 150 rpm using LB culture media containing rifampicin (100 μg/mL) and kanamycin (50 μg/mL). The cultures were pelleted by centrifugation for 10 min at 4000 g, after which the pellets were resuspended in 10 mM MgCl_2_ to reach a final OD_600_ of 0.5. Acetosyringone (4-hydroxy-3,5-dimethoxyacetophenone) was added to each suspension to reach a final concentration of 100 μM for virulence induction, and the suspensions were incubated overnight at 4°C. Agroinfiltration cocktails were prepared by combining the pBIN61 cultures with the silencing suppressor culture (in a 1:1 ratio (v:v)). Solutions were infiltrated into the leaves of 5-week-old wild-type *Nicotiana benthamiana* plants using 5 ml syringes without a needle. The plants were placed in a growth chamber and cultivated for five days post infiltration before harvesting (12 h of light per day, 24°C, 60% relative humidity). For each construct, three plants of two independent biological replicates were used, i.e. six samples per construct.

#### Analysis of the volatile compounds in agroinfiltrated tobacco leaves with DHS-GC–MS

Agroinfiltrated leaves were cut five days after infiltration and immediately placed in a 10 ml hermetically sealed glass flask for DHS-GC–MS analysis. The volatile compounds were analyzed in a Dynamic Headspace DHS GERSTEL® autosampler. The vials were incubated for 10 min at 35°C, to reach sample headspace equilibrium and then connected to a Tenax A trap for 12 min, with 25 ml/min nitrogen flow rate. Analytes were transferred to the GC–MS after thermal desorption in the GERSTEL® Thermal Desorption Unit (TDU) and reconcentration in the Cool Injection System (CIS) in splitless mode with an initial temperature set at 50°C and a final temperature set at 300°C. The volatile compounds were analyzed in an Agilent 5977 gas chromatograph coupled with an Agilent 7890B mass spectrometer (Agilent Technologies, Palo Alto, USA). Loaded trap were desorbed on a DB-WAX polar column (60 m × 0.25 mm, 0.25 μm phase film thickness, Agilent J&W GC column, USA) in splitless mode with the injector temperature set at 250°C. Hydrogen was used as a carrier gas with a fixed flow rate of 1.5 mL/min. The initial oven temperature was 40°C (5 min) and then increased by 2°C/min to 170°C and then by 10°C/min to 250°C where it was maintained for 10 min. The mass spectrometer operated at 70 eV in electron impact (EI) ionization mode. The ionization source was heated at 230°C. After ionization, the molecules were separated according to their mass/charge (m/z) by a quadrupole analyser maintained at 150°C sweeping an interval [40 to 350] m/z in SCAN mode. The set of results was processed using MassHunter Qualitative Analyses software and MS Quantitative Analysis software (version 10.2.1).

## Results

### Sensory characteristics of Geisha Especial derived specialty coffee

**Fig. 1:**
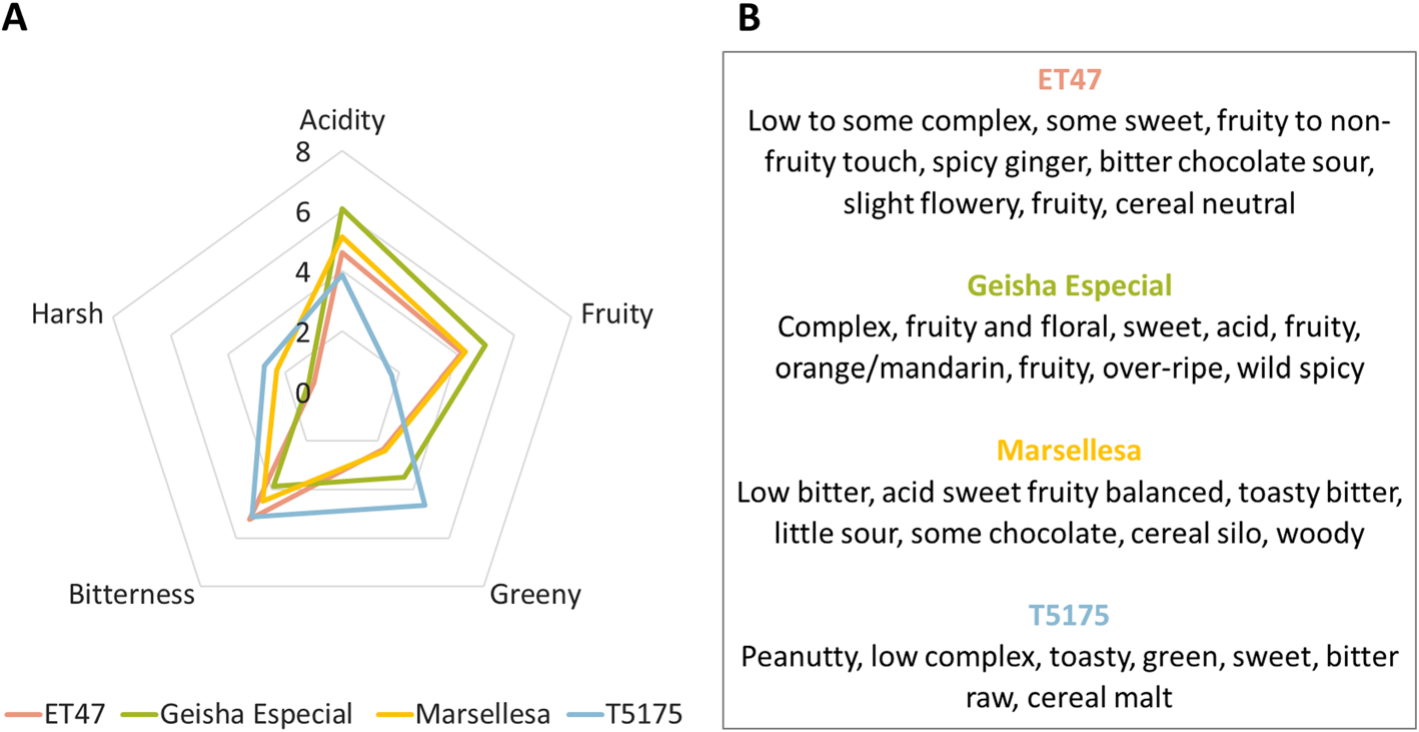
Sensory profile of four Arabica genotypes grown at an elevation of 1300m. **A** Star diagram of five sensory attributes for the four genotypes. Two panels of four judges from two factories (JDE and Nespresso) (*n* = 8) evaluated the samples (mixture of the three trees for each of the four genotypes). The graph shows the score out of 10 (average of the scores given by the 8 judges) for each of the 5 sensory attributes and for each of the 4 genotypes. **B** Qualitative description of the cup's aromatic profile by the 8 judges, for each of the four genotypes

Sensory evaluations were conducted on coffee cups made with roasted beans of the four genotypes of this study and revealed significant differences in sensory perception (Fig. 1A). Geisha Especial obtained the highest scores for the positive attributes (fruitiness and acidity) and the lowest scores for the negative attributes (bitterness and harshness). In addition, the qualitative descriptions only identified the floral and fruity notes that are characteristic of specialty coffees on Geisha Especial derived coffee (Fig. 1B).

The ET47 and Marsellesa genotypes were close in terms of positive attributes, but ET47 had a score close to zero for the harsh attribute, which allowed it to be ranked second behind Geisha Especial in terms of overall quality score. As expected, the lowest scores for positive attributes and the highest ones for negative characteristics were obtained by the T5175 genotype. In more detail, Geisha Especial scored similarly as ET47 in terms of harshness and scored lower than Marsellesa and T5175. In terms of greenness, Geisha Especial scored higher than ET47 and Marsellesa, but lower than T5175. ET47 and Marsellesa had similar profiles, except in the harsh category where Marsellesa scored higher. ET47 and T5175 were the genotypes with the highest bitterness scores.

Both sensory evaluation panels described the Geisha Especial fruity aroma as being an orange/mandarin fruity flavor accompanied by floral notes and a balanced acidity (Fig. 1B). In contrast, T5175 was described as having a low complexity aroma with bitter raw and cereal malt aromas. ET47 was closer to Geisha Especial but less pronounced in terms of fruity and floral character and complexity. Marsellesa was described as having more chocolate notes. These results were consistent with the literature and confirmed that all four genotypes of this study are characterized by unique sensory profiles with Geisha Especial differing most by its citrus fruity notes and balanced acidity.

### Identification of organic volatile substances characteristic of Geisha Especial derived specialty coffee

In order to provide a rationale for the differences of sensory perception of the four Arabica genotypes, quantitative volatilomes of green and roasted beans were established by SPME-GS-MS. Green and roasted beans derived from fresh mature beans (red stage) harvested on 2–3 separate tress (total of 11 samples – 4 genotypes × 2–3 trees).

**Fig. 2:**
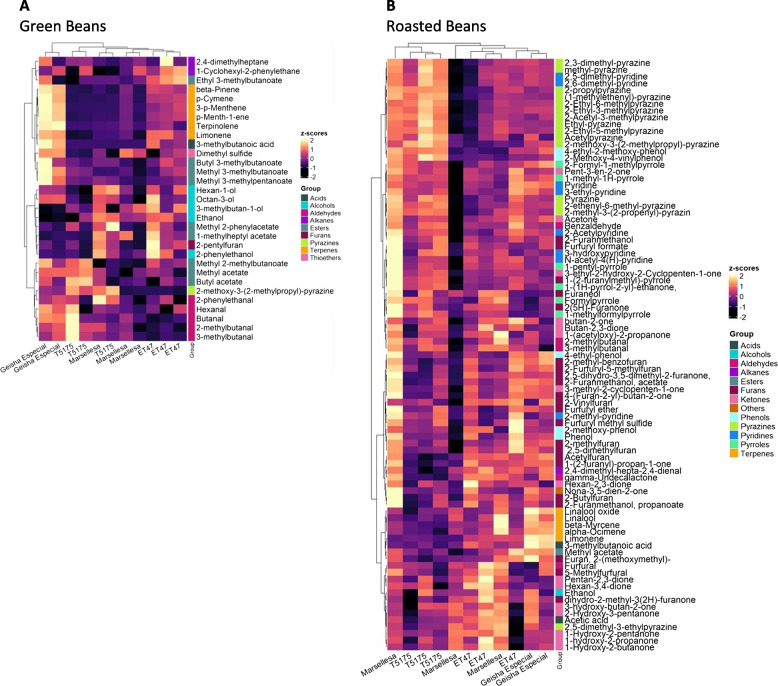
Non-symmetrical heatmap with double hierarchical clustering for **A** green bean volatiles and **B** roasted bean volatiles. The top hierarchical clustering concerned the samples of beans of the different *Coffea arabica* genotypes and the hierarchical clustering on the left-hand side concerned the volatile substances. Heatmap colors show the z-score variation (yellow = high, dark = low) calculated from the log2 transformed semi-quantitative abundance of volatile

Thirty-one volatile compounds were identified in the green coffee bean samples, including (with abundancies) acids (1), alcohols (5), aldehydes (5), alkanes (2), esters (9), furans (1), pyrazines (1), terpenes (6), and thioethers (1) (Fig. 2A). Esters and terpenes were the main groups of volatile compounds identified in the green beans. Hierarchical clustering grouped the samples in two clades with Geisha Especial samples making one of them. This genotype therefore produced mature beans with a significantly different volatile compound profile. ET47 samples also grouped in a unique cluster while tree-to-tree variations did not allow to distinguish Marsellesa and T5175.

Genotype had a significant influence (*p* < 0.01) on 9 of the 31 compounds identified in green beans (Table S5 and Fig. S3). Genotype had a significant influence on all the six monoterpenes as well as on the branched-chain volatile compounds methyl 3-methylbutanoate and 3-methylbutanoic acid, and also on the fatty acid derivative methyl 3-methylpentanoate. For 3-*p*-menthene, *p*-cymene, terpinolene and 3-methylbutanoic acid, Geisha Especial was significantly different from the other 3 genotypes which were assigned to the same group (Tukey's HSD test, *p* < 0.01). Geisha Especial also differed from the other three genotypes for *beta*-pinene and *p*-menth-1-ene. For *beta*-pinene, ET47 was in a different group from Marsellesa and T5175, which were in the same group. For *p*-menth-1-ene, Marsellesa was in an intermediate group between ET47 and T5175, which were different. For limonene, Geisha Especial was significantly different from T5175 and Marsellesa, but Geisha Especial was not significantly different from ET47 despite its higher mean content. Similarly, for methyl 3-methylbutanoate, Geisha Especial was significantly different from Marsellesa and T5175, but not from ET47. For methyl 3-methylpentanoate, Geisha Especial differed from Marsellesa, while T5175 and ET47 were in an intermediate group. Geisha Especial was therefore the most different genotype and was mainly characterized by volatile monoterpenes and branched-chain volatiles. ET47 was in an intermediate group mainly due to limonene, *beta*-pinene, *p*-menth-1-ene and methyl 3-methylbutanoate.

Roasted coffee beans had richer volatile profiles. Eighty-seven volatile compounds were identified, including acids, alcohols, aldehydes, alkanes, esters, furans, ketones, phenols, pyrazines, pyridines, pyrroles and terpenes (Fig. 2B). Roasted beans had retained six of the volatile substances already present in green beans and contained additional volatiles that are oxidation products such as ketones (23 substances) or products of the Maillard and Amadori reactions taking place during roasting such as pyridine and its derivatives (8), pyrazine and its derivatives (15), furan derivatives (18), pyrrole derivatives (6) and furfural derivatives (5). Only one aldehyde could be detected. A correlation heatmap of raosted bean volatile substances contents revealed the existence of clusters of substances suggesting that they may share a common synthetic origin (Fig. S2).

Geisha Especial volatilomes still grouped in a separate clade but were less distinct from those of the other genotypes than during the analysis of green beans. Of the 87 compounds identified in roasted beans, only five were significantly influenced by genotype (*p* < 0.01) (Table S6 and Fig. S3). Interestingly, three of them were already present in green beans (limonene, 2-methoxy-3-(2-methylpropyl)pyrazine and 3-methybotanoic acid). This indicates that roasting affected the green beans of all genotypes in a roughly similar fashion so that roasted beans volatilomes mostly differed because of differences already present in freshly harvested beans. Monoterpenes were strong contributors of roasted beans genetically-derived differences as two of the five discriminating substances were monoterpenes (limonene and linolool oxide) while they only made six of the 87 substances of the global volatilomes. Geisha Especial roasted beans differed from the other three genotypes by containing significantly higher contents of limonene and 3-methylbutanoic acid (Tukey's HSD test, *p* < 0.01). For linalool oxide, Geisha Especial differed from ET47 and T5175, while Marsellesa was in an intermediate group.

In conclusion, roasted beans volatilomes of Geisha Especial differed from those of the other genotypes by having higher contents of limonene and 3-methylbutanoic acid. All of the monoterpenes detected in green and roasted beans are known as flavorings, both in food and in perfumery. Limonene, *beta*-pinene, *p*-cymene, terpinolene, linalool oxide, *beta*-myrcene and linalool are respectively described as citrus, herbal, terpenic, herbal, floral, spicy and floral with regard to the odor category and are respectively described as citrus, pine, terpenic, woody, green, woody and citrus with regard to the flavor category (Table S7 and Fig. S3). The higher limonene content of Geisha Especial in roasted beans is therefore of particular interest because it agrees with the previous sensory evaluations (Fig. 1) that revealed that coffee cups of this genotype are distinguishable from those of the other genotypes by a citrus aroma. Similarly, the more acidic note of Geisha Especial coffee cups correlates with the higher content of the only detected acidic volatile substance of green and roasted beans, 3-methylbutanoic acid.

### Identification of biological and cellular functions associated with Geisha Especial specialty coffee beans

Because bean genotype and ripening stage can both influence bean sensory characteristics, a comparative transcriptomic analysis was conducted on the beans of the four genotypes at two ripening stages (total of 24 RNA samples: two fruit ripening stages x four genotypes x three biological replicates). For this, the 24 cDNA libraries were sequenced by Illumina HiSeq and reads were mapped onto the reference genome *Coffea arabica*: Cara 1.0 genome sequence (Johns Hopkins University). On average, 81% of sequenced reads per sample were uniquely mapped to the reference genome.

**Fig. 3:**
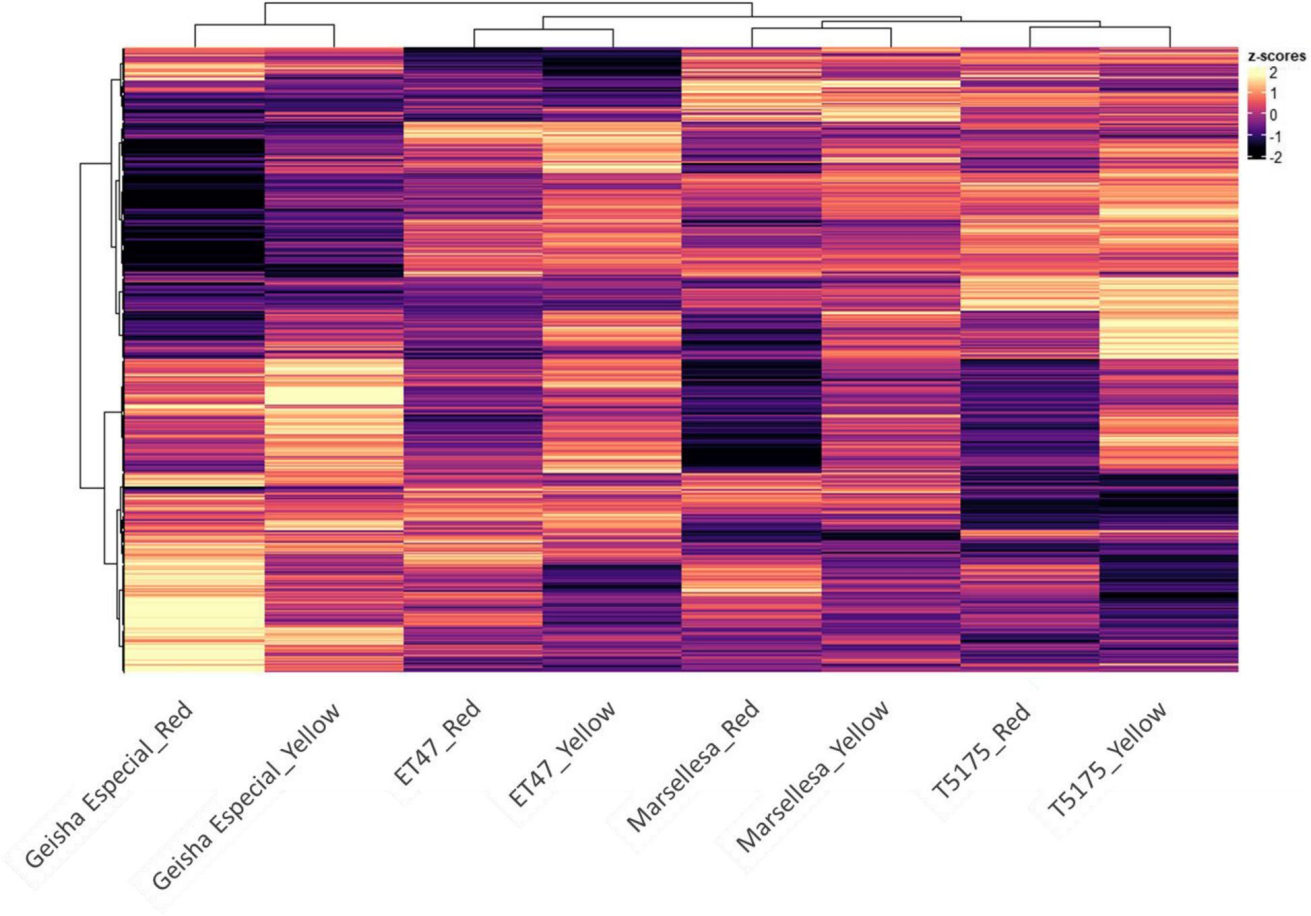
Hierarchical clustering and heatmap of *Coffea arabica* genotypes at both stages of fruit ripening (horizontal axis) and DEGs identified in all the 28 genotype-stage pairwise comparisons (vertical axis). The heatmap represents the z-score calculated from log2 transformed normalized counts (light yellow = up-regulated, dark = down-regulated) (Red for red ripening stage; Yellow for yellow ripening stage)

Based on gene expression levels, a comparative RNA-Seq analysis of the 28 pairwise genotype-stage comparisons was performed. In total, 7 978 genes were selected as being differentially expressed (DEGs—LogFC |1|, FDR < 0.01 in at least one pairwise comparison) and used for a first hierarchical clustering of the four genotypes and their associated ripening stages and a second hierarchical clustering of DEGs enrichment shown on Fig. 3 with DEGs expression levels shown as a heat color. Genotype appeared to be the main driver of this clustering analysis with Geisha Especial samples being most different from the other three genotypes samples in full agreement with the volatile organic compounds (VOCs) comparative analysis conducted above (Fig. 2A). Also, in agreement with the VOCs analysis, the comparative transcriptome analysis supported a differentiation of the three genotypes although ET47 was most different from the others in terms of transcripts while T5175 was most different in terms of VOCs. Ripening stage was a secondary driver of the clustering analysis shown in Fig. 3 as samples of both ripening stages clustered together for each genotype. Interestingly, the parallel hierarchical clustering analysis of DEGs expression levels was clearly driven by the clustering of groups of genes that were over-expressed in specific genotypes. This suggests that each genotype is characterized by the overexpression of a specific set of genes, ripening influencing this over-expression level. A PCA analysis conducted with the same RNA-seq dataset confirmed that Geisha Especial samples differed from the others and that bean ripening influenced transcript levels (Fig. S4).

**Fig. 4:**
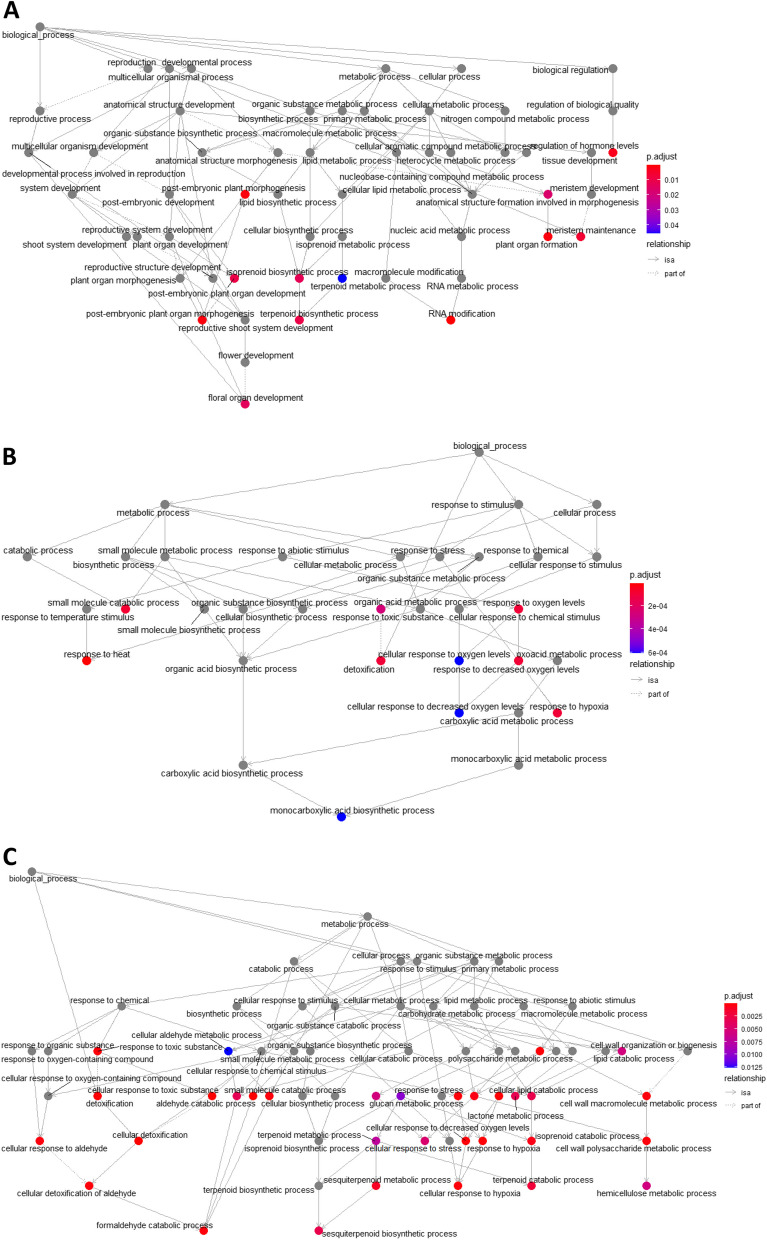
Network representation of a gene ontology enrichment search for biological processes for **A** the 2609 up-regulated DEGs in Geisha Especial (red and yellow ripening stages) over the other cultivars, **B** the up-regulated DEGs in ET47, Marsellesa and T5175 (red and yellow ripening stages) over Geisha Especial, **C** the up-regulated DEGs in yellow ripening stage over the red ripening stage for the all four genotypes. Arabidopsis homologs of up-regulated DEGs were given as input to clusterProfiler R package. The resulting enriched GO terms are visualized using a goplot representation with color-coding for p.adjust value (with threshold set at 0.05), reflecting their degree of enrichment and relationship between each term

To unveil some of the specificity of Geisha Especial, the DEGs that were over-expressed in Geisha Especial samples (at both ripening stages) were filtered. A total of 2 609 genes were identified. A search for gene ontology (GO) enrichment of terms related to biological processes (p.adjust < 0.05) revealed an overrepresentation of genes related to the biosynthetic pathways of isoprenoids (Fig. 4A). This agrees with the higher content of monoterpenes in Geisha Especial beans (Fig. 2). In contrast, GO terms enrichment for the over-expressed genes of the other three genotypes did not highlight metabolic pathways but rather abiotic stress responses to heat and oxygen levels (Fig. 4B). Bean ripening mostly resulted in a lowering of gene expression (LogFC |1|, FDR < 0.01) (Fig. S5). It was most pronounced in Marsellesa where 248 genes were down-regulated during ripening. The difference was also significant for T5175, with 18-times more down-regulated genes as opposed to up-regulated genes (108 genes were down-regulated compared to 6 up-regulated in the red stage compared to the yellow stage). ET47 had 6 down-regulated and 4 up-regulated in the red stage compared to the yellow stage. In Geisha Especial 99 up- and 125 down-regulated genes were observed during ripening. GO terms enrichment for biological processes of up expressed genes (p.adjust < 0.05) in yellow stage beans highlighted an influence of ripening on specialized metabolisms, in particular those associated with the isoprenoid biosynthetic pathway and the small molecules-aldehyde-formaldehyde metabolism (Fig. 4C). Additional effects were seen on cellular lipid catabolism as well as on cell wall polysaccharide metabolic process, the first one being known to feed isoprenoid metabolisms while the second one may relate to fruit softening during ripening.

### Identification of terpene synthases over-expressed in Geisha Especial specialty coffee beans

Among the isoprenoid biosynthetic genes that displayed a higher expression level in the Geisha Especial genotype, 11 genes implicated in the formation of the prenyl-pyrophosphate terpene precursors were identified, including 7 genes belonging to the MEP pathway (Table S8). For these seven genes, genotype had a significant effect on the level of expression (*p* < 0.05). 19 genes were identified as involved in the monoterpenoid (3), sesquiterpenoid (2), diterpenoid (3), triterpenoid (1) and monoterpene indole alkaloid (7) biosynthesis. In addition, 18 genes potentially involved in secondary metabolite oxidation/reduction including 17 cytochromes P450 were identified.

Terpene synthases (TPS) that catalyze the cyclisation step of the terpene carbon backbone play a central, and determinant, role in the blend of terpenes produced by plant [42]. A total of five TPS were more abundantly expressed in Geisha Especial. One was annotated as a (3S,6E)-nerolidol synthase 1-like enzyme (LOC113730856), two as *alpha*-farnesene synthase-like enzymes (LOC113723866 and LOC113723870), one as a *cis*-abienol synthase, chloroplastic-like isoform X1 (LOC113702909) and one as a terpene synthase 10-like enzyme (LOC113729710). The (3S,6E)-nerolidol synthase 1-like enzyme encoded a 572 amino acid protein. Both *alpha*-farnesene synthase-like enzymes encoded 589 and 654 amino acid polypeptides. The *cis*-abienol synthase, chloroplastic-like isoform X1 encoded a 791 amino acid protein. The terpene synthase 10-like enzyme encoded a 606 amino acid protein. Protein sequence alignment of the five TPSs showed high identity between the two alpha-farnesene synthase-like enzymes (93%). In contrast, the other TPSs showed low levels of identity (16–37% identity). All five TPSs possessed the conserved catalytic motifs characteristic of TPSs ‘DDxxD’ and ‘Rxx(N,D)Dxx(S,T,G)xxxE’ (Fig. S6). In contrast, only three of the five TPSs (the two *alpha*-farnesene synthase-like and terpene synthase 10-like) had the 'RxR' motif. In addition, these three TPSs also contained the conserved motif characteristic of TPSs involved in the cyclization of monoterpenes, ‘RRxxxxxxxxW’. Phylogenetic analysis showed that the two genes annotated as farnesene synthase, alpha-farnesene synthase-like (LOC113723866 and LOC113723870), and terpene synthase 10-like (LOC113729710), clustered with the *Vitis vinifera*, *Solanum lycopersicum* and *Arabidopsis thaliana* terpene synthases of the TPS-b clade, which includes the majority of mono-TPSs and those associated with cyclic monoterpene formation (Fig. S7). The other two TPSs lacked the ‘RRxxxxxxxxW’ motif. The absence of this motif leads to the synthesis of acyclic monoterpenes products, such as myrcene or linalool. Phylogenetic analysis indicated that the (3S,6E)-nerolidol synthase 1-like (LOC113730856) clustered with *Vitis vinifera*, *Solanum lycopersicum* and *Arabidopsis thaliana* terpene synthases of the TPS-g subfamily, which includes mono-TPSs associated with acyclic monoterpenes formation. Cis-abienol synthase, chloroplastic-like isoform X1 (LOC113702909) clustered with *Vitis vinifera*, *Solanum lycopersicum* and *Arabidopsis thaliana* terpene synthases of the TPS-e/f subfamily, which includes copalyl diphosphate synthases and kaurene synthases as well as sesqui- and monoterpene synthases.

**Fig. 5:**
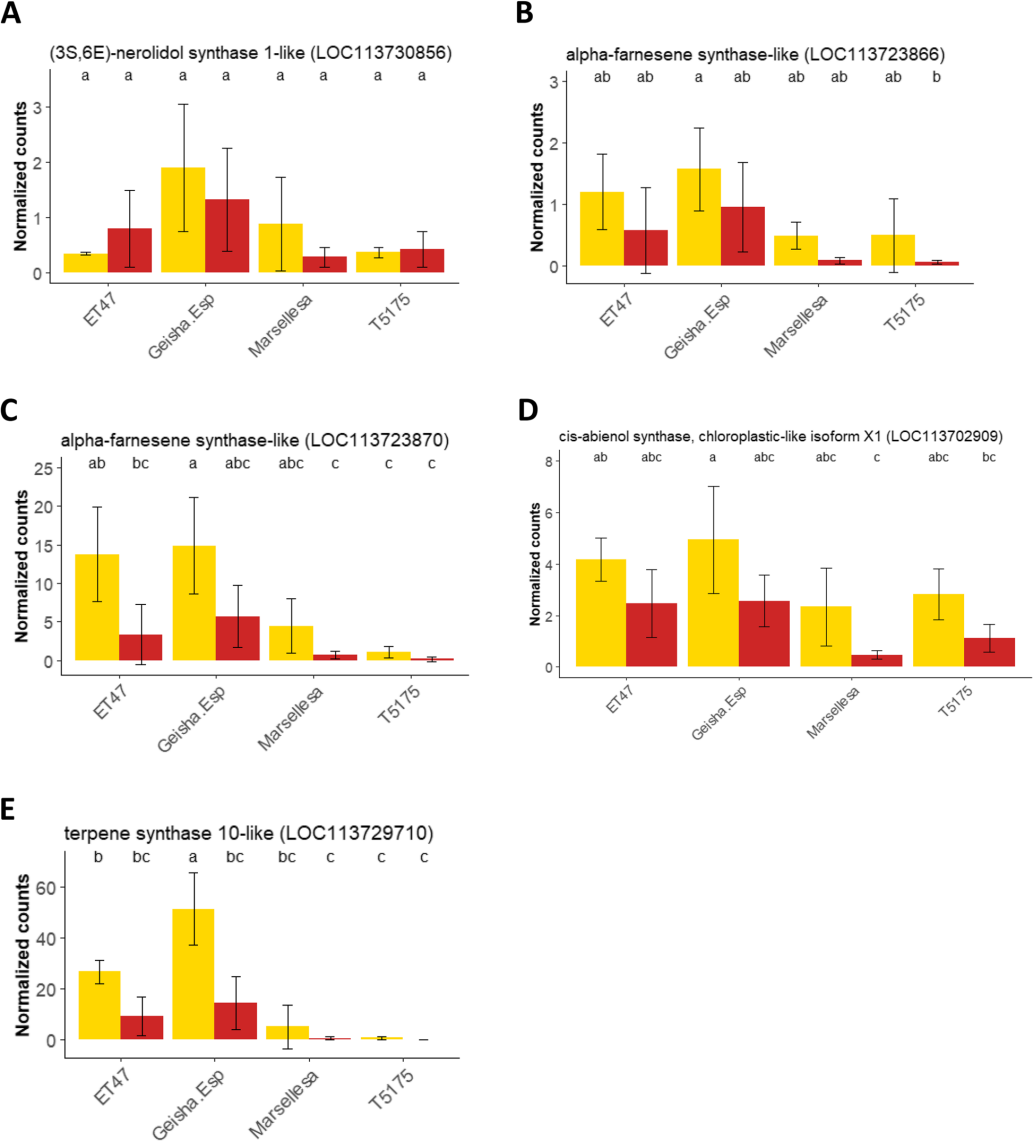
Level of expression (normalized RNA-Seq counts) of terpene synthases for each of the four genotypes at two stages of ripening. Red ripening stage with red bars and yellow ripening stage with yellow bars. Bars represent means + /_ SD of three biological replicates for each condition: genotype x ripening stage. Means with different letters are significantly different (Tukey’s HSD test, *P* < 0.05). **A** (3S,6E)-nerolidol synthase 1-like (LOC113730856), **B** alpha-farnesene synthase-like (LOC113723866), **C** alpha-farnesene synthase-like (LOC113723870), **D** cis-abienol synthase, chloroplastic-like isoform X1 (LOC113702909), **E** terpene synthase 10-like (LOC113729710)

An ANOVA analysis on expression level of the (3S,6E)-nerolidol synthase 1-like enzyme (LOC113730856) in the four genotypes and their two ripening stages suggested that the difference in expression level was not significant among these conditions (Tukey’s HSD test, *P* < 0.05) (Fig. 5A). The expression level of the *alpha*-farnesene synthase-like enzyme (LOC113723866) was significantly different between Geisha Especial yellow stage beans and T5175 in red stage (Fig. 5B) while the expression level of the other *alpha*-farnesene synthase-like enzyme (LOC113723870) was significantly different between Geisha Especial-yellow stage and T5175-yellow stage and T5175, Marsellesa and ET47-red stage (Fig. 5C). The expression of *cis*-abienol synthase, chloroplastic-like isoform X1 (LOC113702909) in Geisha Especial yellow stage was significantly different from the one in Marsellesa and T5175-red stage (Fig. 5D). The fifth identified gene was annotated as a terpene synthase 10-like enzyme (LOC113729710). Unlike the other four TPSs, the expression level of the terpene synthase 10-like enzyme (LOC113729710) matched more closely the accumulation profiles of monoterpenes in beans with significantly higher accumulation in Geisha Especial yellow stage beans compared to all other genotypes and ripening stages (Fig. 5E).

Spearman’s correlation between the expression level of the five terpene synthases and the accumulation levels of the free monoterpenes found in green and roasted beans of the four genotypes were performed (Fig. S8). It revealed that bean monoterpenes contents grouped into two separate positive correlation clusters respectively made of 3-*p*-menthene, *p*-menth-1-ene, *p*-cymene, limonene, *beta*-pinene and terpinolene (cluster one) and linalool oxide, linalool, *beta*-myrcene and *alpha*-ocimene (cluster 2). Only the transcript levels of the terpene synthase 10-like (LOC113729710) positively associated with one of these cluster. Positive correlations were strongest with limonene contents in green and roasted beans.

Protein sequence alignment of the terpene synthase 10-like (*Ca*TPS10-like—XP_027109762.1) isolated from cultivar Arabica Caturra Red with a *C. arabica* cv. Catuai Red terpene synthase (*Ca*TPS1—CCM43927.1) functionally characterized as a limonene synthase [13] revealed a high level (93%) of identity (Fig. S9) (nucleotide sequence alignment revealed 96% of identity – Fig. S10). This latter enzyme was described to be expressed in coffee drupes 25 weeks after pollination (corresponding to theoretical stage 5, when the pericarp is still green) and absent from fully ripe drupes or coffee beans [13] unlike the terpene synthase 10-like protein. *Ca*TPS10-like protein contained an extra 40 amino acids long N-terminal stretch compared to *Ca*TPS1. The N-terminal part of *Ca*TPS1 was predicted to act as either a mitochondrial targeting peptide or a chloroplast targeting peptide [13]. With its longer extension, the N-terminal sequence of *Ca*TPS10-like was predicted to be a chloroplast targeting peptide (70% probability) by the Target P address prediction software [43]. The presence of an N-terminal chloroplast targeting signal peptide is a general characteristic of most monoterpene synthases in agreement with the biosynthetic origin and greater abundance of their geranyl pyrophosphate substrate in chloroplasts [44]. Apart from this addressing peptide, both sequences were identical with the exception of a single amino acid change (R422L for *Ca*TPS10-like).

### Functional characterization of *Ca*TPS10-like enzyme

To obtain direct evidence for the function of the terpene synthase 10-like (*Ca*TPS10-like) enzyme, transient expression of the full gene sequence was performed in *N. benthamiana* leaves. Parallel transfections were conducted with two other mono-TPS known to be involved in producing limonene, *Ca*TPS1 (35S:TPS1 – full gene sequence), a limonene synthase characterized in *C. arabica* fruits [13] and *Sl*TPS7 (35S:TPS7), a *beta*-Myrcene/Limonene synthase characterized in *S. lycopersicum* fruits [40]. Transfection with the empty vector (35S:pBIN61) was used as a negative control.

**Fig. 6:**
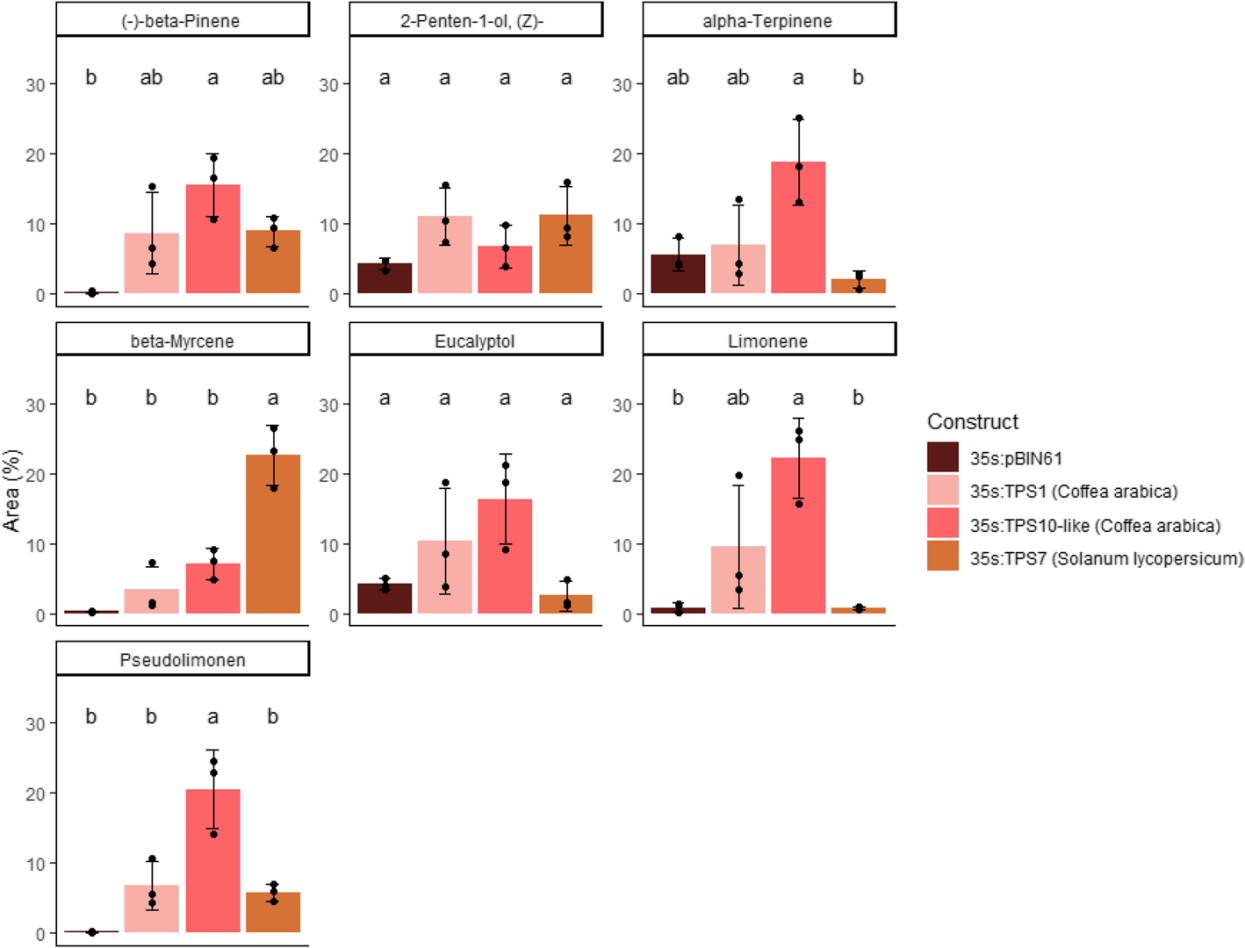
DHS-GC–MS analyses of the *N. benthamiana* leaves volatile compounds following transient expression. *N. benthamiana* leaves were transformed with terpene synthase 10-like (35S:TPS10-like (*Coffea arabica*)) and two other mono-TPSs that have been shown to be involved in limonene production. TPS1 (35S:TPS1 (*Coffea arabica*)) was characterised as a limonene synthase in *Coffea arabica* by Del Terra et al. [13]. It was found to be expressed in drupes 25 weeks after pollination and was used here as a positive control. TPS7 (35S:TPS7 (*Solanum lycopersicum*)) was characterised as a *beta*-myrcene/limonene synthase in *Solanum lycopersicum* by Zhou & Pichersky [40]. Leaves of *N. benthamiana* were transformed with a TPS construct (35:TPS10-like, 35S:TPS1, 35S:TPS7) or with the 35S:pBIN61 empty vector for the negative control. For each construct, three independent biological replicates were used to quantify relative amounts of volatile compounds. Relative amounts are expressed as mean percentage of total peak areas; individual values are indicated with black dots; bars indicate the standard error. Means with different letters are significantly different (Tukey’s HSD test, *P* < 0.01)

Analysis of the volatiles compounds emitted by infiltrated leaves five days after infiltration revealed the presence of six monoterpenes ((-)-*beta*-pinene, pseudolimonene, *beta*-myrcene, limonene, eucalyptol, *alpha*-terpinene and 2-Penten-1-ol, (Z)-) (Fig. 6 and Fig. S11). Since 2-penten-1-ol, (Z)- was detected in leaves infiltrated with the empty vector, its presence was not linked to the ectopic expression of the TPSs. The leaves transformed with *Ca*TPS1 emitted the same blend of volatile substances as those expressing *Sl*TPS7 (Student’s t test, *P* ≤ 0.05). The leaves expressing *Ca*TPS10-like emitted significantly greater amounts of *alpha*-terpinene and pseudolimonene than those expressing the two other TPSs. It emitted non-significantly different levels of limonene compared to *Ca*TPS1 but its limonene emission was greater than in leaves expressing *Sl*TPS7. *Ca*TPS10-like is therefore a limonene synthase that produces mostly limonene and minor quantities of (-)-*beta*-pinene and pseudolimonene.

## Discussion

The coffee industry is at a turning point of its history. Until recently, emphasis was placed on the stimulatory effect (caffeine) and stringency of the coffee drink. An early differentiation of products was based on the caffeine content (Arabica vs Robusta), a differentiation that actually mostly reflects an inter-specific difference within the genus *Coffea* [45]. Secondary differences in pre- and post-harvest treatments (from fermentation and roasting) were also valued to differentiate market produce with different cupping evaluations [46–50] as is commonly done for tea, another stimulatory hot drink [51]. Only recently has interest shifted to coffee tree variety-specific aroma to differentiate the current market into sub-categories. The typical aroma of the roasted beans of the coffee variety *Coffea arabica* var. Laurina, a specialty coffee known as 'Bourbon Pointu' was associated with the presence of 22 compounds with fruity, chocolate and caramel aromas [52]. Within this variety, Grand Cru coffee roasted beans had enhanced fruity notes and were richer in unsaturated aldehydes such as (E,E)-2,4-nonadienal and (E,Z)-2,4-heptadienal [52]. A differentiation of the biochemical composition and quality of roasted coffee beans of 12 Arabica Bourbon-Typica and Arabica introgressed cultivars showed that genetic characteristics have a major influence on the chemical composition [53]. Nevertheless, to the best of our knowledge, the present study is the first one that aimed to link a *C. arabica* variety-specific ripe bean gene expression trait to the coffee cup aromatic characteristic. The variety Geisha Especial could thus be differentiated from a group of three other varieties by generating coffees with a more citrus and acidic flavor, the citrus note being linked to higher limonene contents, a substance that was in higher content in green beans due to the higher expression of its biosynthetic genes in the beans of ripe berries.

Mass production of coffee beans relies on the worldwide cultivation of very few genotypes. The need to adapt the crop to climate change and disease pressures has led scientists and breeders to explore the genetic diversity of coffee species [54]. With the current efforts to conduct sensory evaluation of the newly developed coffee varieties, it will be interesting to see whether the citrus note found in Geisha Especial ripe beans is the sole fruity note that can affect specialty coffee aroma or whether a larger panel of variety-derived aromas exists as exemplified by winemaking grapevine cultivars [55]. The terpene biosynthetic pathway may deserve special interest as it is largely unknown in *C. arabica* and is a source of diverse aromas. The present study also revealed that the TPS gene family is diversified in *C. arabica* and that the entire terpene gene biosynthetic pathway may be up-regulated in a specific variety such as Geisha Especial.

Volatile organic compounds constitute a diverse family of substances that play a major role in taste perception of food and beverages. Volatile phenylpropanoids, methoxypyrazines, branched chains amino acid derivatives and terpenoids are thus known to be major contributors of the aroma of cocoa, strawberries, grapes and tomatoes for example [55–60]. Out of the multitude of volatile compounds emitted by plant produce, only a few are often prevailing and dominate taste perception of a given produce. As these may be specific to certain genotypes within a genus, they are used to define intraspecific chemotypes. For example, out of the 400 volatile compounds that have been isolated from the tomato fruit, only 21 have been identified as major contributors to the quality of flavor: mainly terpenes, phenylpropanoids, lipids and branched-chain amino acids [60]. In apple, about 350 volatile compounds have been identified [61], from which only about 20, including the sesquiterpene farnesene, are considered important to distinguish the flavors of different apple varieties [62, 63]. Similarly, over 300 volatile compounds have been identified in mature strawberry fruit, and only a few of these can be used to differentiate cultivated and wild accessions [56].

In Arabica coffee, few studies have identified green bean volatile compounds important for coffee beverage flavor despite extensive descriptions of the rich blend of volatile compounds of green and roasted beans [46]. Glycosylated precursors of 3-methylbutanoic acid were found to be associated with high cupping scores in green beans [64]. Sixteen different batches of roasted Guatemalan coffee beans differed in 3-methylbutanoic acid methyl ester content, a substance that was associated with the fresh, fruity aroma and cleanliness of the coffee [65]. The results of our study are in line with these finding as the volatile compound 3-methylbutanoic acid was found to be at a higher content in both green and roasted beans of the Geisha Especial genotype. Some green bean monoterpenes were suggested to participate in the final aromatic bouquet of coffees [13] and improve the quality of the beverage [66]. Our data confirm this hypothesis and suggest that limonene is the monoterpene that mostly contributes to coffee cup quality. In green coffee beans, we found that Geisha Especial green beans are characterized by the higher abundance of six monoterpenes (3-*p*-menthene, *beta*-pinene, *p*-menth-1-ene, limonene, *p*-cymene and terpinolene). However, terpenic compounds degrade with increasing roasting temperature [66]. Geisha Especial roasted beans volatile terpenes therefore unsurprisingly differed by a different mix of monoterpenes which were linalool oxide, limonene, linalool and *β*-myrcene. Limonene therefore was the only green bean monoterpene that survived roasting. The other monoterpenes present in the roasted beans were probably formed from green bean monoterpenes during the roasting process. Myrcene is indeed industrially produced by pyrolysis of *β*-pinene [11] so that green bean *β*-pinene may be the source of roasted bean myrcene. The fact that the monoterpenes identified in the roasted beans were less discriminating than those identified in the green beans may be related to their degradation during the roasting process. Interestingly, the vast majority of roasted bean volatiles that derived from the Maillard and Amadori thermal reactions of non-terpene primary metabolite such as sugars and amino acids did not distinguish coffee cups made from different roasted bean genotypes. Coffee is not the first example of a plant species where its volatile monoterpenes are major determinants of genotype specificity in transformed beverage products. The typical fruity and floral notes of Muscat and Gewürztraminer wines is linked to a unique blend of volatile monoterpenes in the ripe grapes [55, 67]. Some monoterpenes are also characteristic of the fine cocoa genotype SCA6 [58] and are related to the floral note of Nacional cocoa [68]. Brewed coffee aroma volatiles were not analyzed in this study. However, limonene has been identified in espresso coffee by several studies [14, 16, 17] and is known to be extracted by 98°C hot water during the preparation of hibiscus flower infusions [69]. Brewing is nonetheless a multi-parameter process where minute differences can greatly affect the extraction of volatile compounds and may result in some degree of terpene glycoside hydrolysis. Aroma perception is also very complex as it is subject to matrix retention and synergistic effects among many compounds for perception. Sensory-focused studies are therefore now needed to define aroma transfer mechanisms from roasted beans to the coffee beverage and to establish the threshold perception levels for all coffee aroma compounds. Terpene synthesis is known to be under the control of both developmental and environmental cues [70]. In this study, transcriptome comparison between the yellow and red stages of ripening beans (last two stages of ripening) revealed few differentially expressed genes (DEGs). A comparison of the transcriptomes of *C. arabica* cv. K7 beans at the last three stages of ripening (green, yellow and red) revealed also the lowest number of DEGs when comparing the last two stages of ripening [71]. Only 130 DEGs were identified by these authors between the red and yellow stages. Similarly, very few DEGs were identified during this last step of ripening for the three studied species, *C. arabica*, *C. eugenioides* and *C. canephora* [72]. Interestingly, we found that the terpene pathway follows this trend as it was found to be more highly expressed at the yellow stage than at the red stage. This could indicate that terpenes are predominantly formed at the yellow stage in agreement with an earlier observation [73] that showed a decreased expression of the early terpene biosynthetic gene DXR during the final stage of fruit development. Similarly, it was found that the phenylpropanoid pathway was more expressed at the yellow stage [71]. Nevertheless, even though all studied *C. arabica* genotypes followed the same developmental trend, they differed in their level of expression of the members of the terpene pathway, suggesting that genotype specific differences underpin the strength of volatile terpene biosynthesis in *C. arabica* in agreement with earlier suggestions from Silva et al. 2020 [73].

Terpene synthases (TPS) catalyse the formation of the carbon backbone of terpenes and, as such, commit terpenes biosynthesis into the different terpene subclasses [42]. Unsurprisingly, among the very few genes which expression paralleled the volatile monoterpene emission, five were TPS. Among them, only one (*Ca*TPS10-like) had an expression pattern that correlated among genotypes with the emission of limonene, the volatile monoterpene most closely associated with a fruity aroma in coffee beverages. Its expression level also decreased during the last ripening stage. Transient ectopic expression of *Ca*TPS10-like (isolated from cultivar Caturra Red) confirmed that this TPS acts as a limonene synthase and further indicated that it is responsible for the co-synthesis of *β*-pinene and pseudolimonene, substances that also accumulated in greater amounts in the green beans of the specialty genotype Geisha Especial but were destroyed, or maybe transformed into other monoterpenes, during roasting.

Previously, a limonene synthase from *C. arabica* (*Ca*TPS1) was described [13]. Its protein sequence is similar to that of *Ca*TPS10-like, except for a shorter N-terminal sequence. This sequence is predicted to be a mitochondria/chloroplast-targeting peptide for *Ca*TPS1 and a chloroplast-targeting peptide for *Ca*TPS10-like. Additionally, a lysine residue at position 422 has been substituted by an arginine in *Ca*TPS10-like. Both gene sequences originated from different genotypes (Catuai Red for *Ca*TPS1 and Cattura Red for *Ca*TPS10-like). Our transient heterologous expression of both complete gene sequences in *N. benthamiana* indicated that they share the same function of mostly producing limonene. This suggests that both predicted targeting peptides are functional as both enzymes used GPP as a substrate (GPP is the substrate of monoterpenes and is mostly found in chloroplasts) and that the L4222R mutation is silent. Despite this varietal gene difference being silent in terms of function, the expression pattern of both genes differed during fruit ripening. [13] *Ca*TPS1 was found to be expressed in drupes at earlier stage of ripening (25 weeks after pollination, corresponding to theoretical stage 5, when the drupe pericarp is still green) [13] while *Ca*TPS10-like was mostly expressed in later stages of ripening (this study). Unfortunately, insufficient genome sequences of *C. arabica* are available to know whether both genes represent different alleles of the same locus or paralogous genes.Two additional monoterpene synthases, *Ca*TPS2 and *Ca*TPS3, have also been described [13]. Both were expressed in flowers and fruits at earlier stages of bean development. Both synthesized L-linalool and *β*-myrcene. None of them was expressed in mature coffee bean and we did not see their expression in yellow and red coffee beans. However, we unveiled that four other TPSs are expressed in ripe beans. These were not functionally analyzed because their expression level did not strictly correlate with limonene content, the trait associated with the greater quality appreciation of the Geisha Especial genotype. Nevertheless, this result and previous studies [13] suggest that a wide variety of TPS are expressed in ripening coffee beans. This opens the possibility that differences in their function and expression among *C. arabica* genotypes may lead to differences in bean aroma with potential impact on coffee cup appreciation in addition to the citrus note described in this study for the specialty genotype Geisha Especial.

## Conclusions

This study is the first one to suggest a causal link between a *Coffea* genomic trait and the higher taste appraisal of a specialty coffee genotype. The greater appreciation of the coffee beverage made from the specialty coffee genotype Geisha Especial was first associated with a citrus and acidic aroma and the greater content in roasted and fresh beans of the monoterpene limonene known to possess citrus flavor and of 3-methylbutanoic acid. Gene ontology searches of genes displaying a greater expression in Geisha Especial freshly harvested beans clearly pinpointed the terpene biosynthetic pathway, a conclusion confirmed by the greater expression of the prenyl-pyrophosphate precursors of terpenes and of five terpene synthases responsible for the making of the terpene carbon skeleton. Among them, only one, *Ca*TPS10-like, had an expression pattern that correlated with limonene accumulation. Its functional analysis confirmed that it acts as a limonene synthase. All in all, *Ca*TPS10-like greater expression in ripening coffee beans of the genotype Geisha Especial was associated with the more fruity flavor of the coffee beverages made from the specialty coffee genotype Geisha Especial. This information will be useful to breeders and growers to respectively hasten specialty coffee genotypes selection and improve growing practices that yield beans with improved taste properties. Given that environmental conditions, particularly temperature, are known to affect the aroma profile of coffee [74], studying the regulation of terpene synthase expression as a function of environmental factors is now a priority to respond to the challenges facing the industry in the face of global change.

### Supplementary Information

**Supplementary Material 1.**

## Data Availability

Data sheets necessary to reproduce the analyses have been deposited in the CIRAD Dataverse and are available under https://doi.org/10.18167/DVN1/NHCK5F. Raw sequence data have been deposited in the European Nucleotide Archive with the primary accession code PRJEB71094. Raw GC–MS files have been deposited in the MetaboLights repository with the primary accession code MTBLS9183.
