# Supplementary material for: Combined sensory, volatilome and transcriptome analyses identify a limonene terpene synthase as a major contributor to the characteristic aroma of a Coffea arabica L. specialty coffee
Source: BMC Plant Biol. 2024 Apr 3;24:238. doi: 10.1186/s12870-024-04890-3 (PMC10988958; doi:10.1186/s12870-024-04890-3)
Supplement: Supplementary file 1 — Supplementary Material 1. [file 12870_2024_4890_MOESM1_ESM.docx]

**Additional file**

# **Title**

Combined sensory, volatilome and transcriptome analyses identify a limonene terpene synthase as a major contributor to the characteristic aroma of a *Coffea arabica* L. specialty coffee

# **Authors**

Lison Marie^a*^, Jean-Christophe Breitler^a^, Pingdwende Kader Aziz Bamogo^b^, Mélanie Bordeaux^c^, Séverine Lacombe^b^, Maëlle Rios^b^, Marc Lebrun^d^, Renaud Boulanger^d^, Eveline Lefort^a^, Sunao Nakamura^e^, Yudai Motoyoshi^e^, Delphine Mieulet^a^, Claudine Campa^f^, Laurent Legendre^g^, Benoît Bertrand^a^

**Affiliations**

^a^ CIRAD, UMR DIADE, 911 Avenue Agropolis, BP 64501, 34394 Montpellier, France

^b^ IRD, UMR PHIM, 911 Avenue Agropolis, BP 64501, 34394 Montpellier, France

^c^ FUNDACION NICAFRANCE, Matagalpa, Nicaragua

^d^ CIRAD, UMR QualiSud, TA B95/16 - 73 rue Jean-François Breton, 34398 Montpellier Cedex 5, France

^e^ Research Institute, Suntory Global Innovation Center Limited, 8-1-1, Seika-dai, Seika-cho, Soraku-gun, Kyoto, 619-0284, Japan

^f^ IRD, UMR DIADE, 911 Avenue Agropolis, BP 64501, 34394 Montpellier, France

^g^ INRAE, UR 1115 Plantes et Systèmes de Culture Horticoles, Site Agroparc, 84914 Avignon, France

* Corresponding author: E-mail, lison.marie@ird.fr

L. Marie; CIRAD, UMR DIADE, Université de Montpellier, IRD, CIRAD, 911 Av. Agropolis, 34394 Montpellier, France, +33 467416273, lison.marie@ird.fr

**Table S1.** Coffee cup tasting: example of a scoring grid for a judge. Each tasting is blind. The order of tasting is different for each judge. To mask the identity of the sample, each sample was labelled with a letter a-k. The judge tastes the coffees in the order of the grid and scores each descriptor on a scale from 0 to 10. Coffee preparation protocol: Grind 20 g of coffee per sample to a medium to fine powder; Weigh 15 g* of coffee powder into each piston coffee maker (Bodum 500ml French press); Bring the water to the boil in the electric kettle; Measure out 300 mL* in a test tube and pour into each coffee maker (the coffee will rise to the top of the jug); Stir the coffee with a spoon to circulate and homogenise the drink; Leave to brew for exactly five minutes; Filter the drink with the plunger of the coffee machine; Pour the coffee (60-70 mL) into each cup (4 cups in total); The tasting should take place at a temperature of between 50 and 55 °C.

* ISO standard NF 6668 recommends using 7 ± 0.1 g of coffee per 100 mL of water. However, it states that other quantities may be used. In the CIRAD laboratory, a ratio of 5 g per 100 mL of water is preferred because it delays the onset of sensory saturation in the judges.

|  | | **Series 1** | | | **Series 2** | | |  | **Series 3** | | | **Series 4** | |
| --- | --- | --- | --- | --- | --- | --- | --- | --- | --- | --- | --- | --- | --- |
|  | | **1** | **2** | **3** | **4** | **5** | **6** | **Break** | **7** | **8** | **9** | **10** | **11** |
|  |  | **c** | **b** | **g** | **e** | **d** | **j** |  | **f** | **k** | **h** | **i** | **a** |
| **TASTE** | **Acidity** |  |  |  |  |  |  |  |  |  |  |  |  |
|  | **Bitterness** |  |  |  |  |  |  |  |  |  |  |  |  |
| **AROMA** | **Fruity** |  |  |  |  |  |  |  |  |  |  |  |  |
|  | **Harsh** |  |  |  |  |  |  |  |  |  |  |  |  |
|  | **Greeny** |  |  |  |  |  |  |  |  |  |  |  |  |
| **Comments** | |  |  |  |  |  |  |  |  |  |  |  |  |

**Table S2.** Green coffee beans volatile compounds identification. Black stars indicate molecules previously identified with a standard. Retention times (RT), Experimental retention indices of Kovats calculated on a DB-WAX polar column (60 m × 0.25 mm, 0.25 μm phase film thickness, Agilent J&W GC column, USA) (RI exp.), Match factor of MS spectrum (Match Factor), NIST library RI (polar) reference values, Molecules previously identified in green coffee beans (References). The chirality of enantiomeric substances was not determined so that substances such as limonene are either (+)-limonene or (-)-limonene.

| Volatile compounds | RT | RI exp. | Match Factor | NIST library RI | References^1^ |
| --- | --- | --- | --- | --- | --- |
| Dimethyl sulfide * | 3.187 | 799 | 81.8 | 753 | a, b |
| 2,4-dimethylheptane | 3.629 | 823 | 85.3 | 797 |  |
| Methyl acetate * | 3.766 | 830 | 92.6 | 828 | b |
| Butanal | 4.349 | 859 | 81.5 | 877 | c |
| 2-methylbutanal * | 4.976 | 888 | 74 | 914 |  |
| 3-methylbutanal * | 5.057 | 892 | 70.5 | 918 | b, f, j |
| Ethanol * | 5.6 | 916 |  | 932 | b, i, j |
| Methyl 2-methylbutanoate | 7.617 | 996 | 92.7 | 1009 | h |
| Methyl 3-methylbutanoate | 7.971 | 1008 | 84.3 | 1019 | h |
| 1-Cyclohexyl-2-phenylethane | 8.624 | 1030 | 73 |  |  |
| 3-*p*-Menthene | 9.672 | 1062 | 93.6 | 1078 |  |
| Ethyl 3-methylbutanoate | 10.011 | 1071 | 91 | 1068 | b, h |
| Butyl acetate * | 10.163 | 1075 | 83.2 | 1074 | g |
| Hexanal * | 10.465 | 1084 | 88.2 | 1083 | b, f |
| *beta*-Pinene | 11.159 | 1102 | 86.8 | 1112 | i |
| *p*-Menth-1-ene * | 12.619 | 1135 | 94.8 | 1150 |  |
| Methyl 3-methylpentanoate | 12.907 | 1142 | 68.8 | 1129 |  |
| 1-Butanol * | 14.3 | 1169 | 97.4 | 1142 | / |
| Limonene * | 16.397 | 1204 | 97.6 | 1199 | f, i |
| 3-methylbutan-1-ol * | 17.828 | 1224 | 77.4 | 1209 | a, j |
| 2-pentylfuran | 18.621 | 1234 | 74 | 1232 | b |
| Butyl 3-methylbutanoate | 20.093 | 1252 | 90.7 | 1259 |  |
| *p*-Cymene | 20.846 | 1260 | 89.9 | 1272 |  |
| Terpinolene * | 21.598 | 1269 | 83.5 | 1284 |  |
| 1-methylheptyl acetate | 22.092 | 1274 | 63 |  |  |
| Hexan-1-ol * | 26.962 | 1323 | 88.2 | 1355 | a, b, e |
| Octan-3-ol | 29.633 | 1350 | 62.9 | 1393 |  |
| 2-methoxy-3-(2-methylpropyl)-pyrazine | 37.022 | 1437 | 83.2 | 1518 | a, e |
| 2-phenylethanal | 43.035 | 1517 | 73.1 | 1641 | f, j |
| 3-methylbutanoic acid * | 45.528 | 1548 | 87 | 1666 | a, d, i, j |
| Methyl 2-phenylacetate | 49.918 | 1592 | 74 | 1750 |  |
| 2-phenylethanol * | 57.266 | 1596 | 81.3 | 1907 | a, f |

^1^ a = Bertrand et al. 2012 ; b = Gonzalez-Rios et al. 2007a ; c = Hadj Salem et al. 2020 ; d = Holscher and Steinhart 1995 ; e = Lee et al. 2002 ; f = Lee et al. 2017a; g = Pereira et al. 2015 ; h = Scheidig et al. 2007 ; i = Vezzulli et al. 2023 ; j = Yeretzian et al. 2002

**Table S3.** Roasted coffee beans volatile compounds identification. Black stars indicate molecules previously identified with a standard. Retention times (RT), Experimental retention indices of Kovats calculated on a DB-WAX polar column (60 m × 0.25 mm, 0.25 μm phase film thickness, Agilent J&W GC column, USA) (RI exp.), Match factor of MS spectrum (Match Factor), NIST library RI (polar) reference values, Molecules previously identified in roasted coffee beans (References). The chirality of enantiomeric substances was not determined so that substances such as limonene are either (+)-limonene or (-)-limonene.

| Name | RT | RI exp. | Match Factor | NIST library RI | References^1^ |
| --- | --- | --- | --- | --- | --- |
| Acetone * | 3.641 | 837 | 89.8 | 819 |  |
| Methyl acetate * | 3.766 | 844 | 92.6 | 828 | f |
| 2-methylfuran * | 4.253 | 869 | 88.7 | 870 | b, d |
| butan-2-one * | 4.7 | 891 | 99.5 | 907 | f |
| 2-methylbutanal * | 4.969 | 903 | 86.7 | 914 | f |
| 3-methylbutanal * | 5.057 | 907 | 88.3 | 918 | b, d |
| Ethanol * | 5.6 | 931 |  | 932 | d |
| 2,5-dimethylfuran | 5.88 | 942 | 92.5 | 939 | d |
| Butan-2,3-dione * | 6.6 | 970 | 87.4 | 979 | b, d |
| Pentan-2,3-dione * | 9.55 | 1062 | 88.4 | 1058 | b, d |
| 2-Vinylfuran | 10.063 | 1075 | 72.9 | 1075 |  |
| Pent-3-en-2-one * | 12.601 | 1130 | 63.2 | 1128 |  |
| Hexan-2,3-dione | 12.97 | 1137 | 77.1 |  |  |
| 1-methyl-1H-pyrrole | 13.154 | 1140 | 81.7 | 1145 | b |
| Hexan-3,4-dione | 13.479 | 1146 | 71.8 |  | c |
| 1-Butanol * | 14.147 | 1158 | 68.7 | 1142 | / |
| *beta*-Myrcene * | 14.8 | 1169 | 87.9 | 1161 | a |
| Pyridine * | 15.777 | 1184 | 95.8 | 1186 | b, d, f |
| Limonene * | 16.5 | 1195 | 78.3 | 1199 | d, e, f |
| Pyrazine | 17.36 | 1208 | 67.9 | 1212 | b, d, f |
| 2-methyl-pyridine | 18.079 | 1218 | 72.7 | 1220 | d |
| Furan, 2-(methoxymethyl)- * | 19.104 | 1233 |  | 1247 | f |
| *alpha*-Ocimene * | 20.086 | 1246 | 72.9 | 1245 |  |
| dihydro-2-methyl-3(2H)-furanone | 20.521 | 1252 | 92.1 | 1268 |  |
| methyl-pyrazine * | 20.743 | 1255 | 91.1 | 1266 | f |
| 3-hydroxy-butan-2-one * | 21.801 | 1269 | 81.3 | 1285 | b, d |
| 1-hydroxy-2-propanone * | 22.653 | 1280 | 79.3 | 1303 | d |
| 2,5-dimethyl-pyridine * | 24.343 | 1300 | 86.8 | 1370 | b, d, f |
| 2,6-dimethyl-pyridine * | 24.749 | 1305 | 86.8 | 1258 | b, d, f |
| Ethyl-pyrazine * | 25.014 | 1308 | 94.6 | 1337 | b, d, f |
| 2,3-dimethyl-pyrazine * | 25.815 | 1318 | 91.2 | 1344 | b, d, f |
| 2-Hydroxy-3-pentanone * | 26.508 | 1325 | 67 | 1366 |  |
| 3-methyl-2-cyclopenten-1-one | 26.859 | 1329 | 80.3 | 1507 | d |
| 1-Hydroxy-2-butanone * | 27.334 | 1334 | 74 | 1388 | b, d |
| 3-ethyl-pyridine | 28.128 | 1342 | 86.4 | 1378 | b, d |
| 2-Ethyl-6-methylpyrazine * | 28.371 | 1345 | 91.8 | 1386 | b, d, f |
| 2-Ethyl-5-methylpyrazine * | 28.729 | 1348 | 92.4 | 1387 | b, d, f |
| 2-Ethyl-3-methylpyrazine | 29.581 | 1356 | 85.8 | 1406 | b, d |
| 2-propylpyrazine | 30.315 | 1362 | 81.5 | 1430 | e |
| 1-Hydroxy-2-pentanone | 31.754 | 1372 | 65.7 | 1457 |  |
| Acetic acid * | 31.994 | 1373 | 96.5 | 1449 | b, d, f |
| 2,5-dimethyl-3-ethylpyrazine | 32.252 | 1374 | 74.3 | 1443 | f |
| Furfural * | 32.8 | 1377 | 81.4 | 1461 | b, d |
| 1-(acetyloxy)-2-propanone * | 33.425 | 1379 | 89.1 | 1474 | d |
| Linalool oxide * | 33.9 | 1381 | 88.2 | 1444 | e |
| Furfuryl methyl sulfide | 34.281 | 1382 | 88.4 | 1489 | b, d |
| 2-ethenyl-6-methyl-pyrazine | 34.546 | 1382 | 79 | 1490 |  |
| Furfuryl formate | 35.155 | 1383 | 67.3 | 1501 | d |
| Acetylfuran | 35.343 | 1383 |  | 1499 | b, d |
| Benzaldehyde * | 36.066 | 1382 | 66.6 | 1520 | d, f |
| 2-Butylfuran | 36.391 | 1381 | 77.6 | 1123 | e |
| 2-methoxy-3-(2-methylpropyl)-pyrazine | 37.044 | 1379 | 66.3 | 1518 |  |
| 2-Furanmethanol, acetate * | 37.678 | 1376 | 96.7 | 1533 | g |
| Linalool * | 38.873 | 1368 | 81.9 | 1547 | f |
| 5-Methylfurfural * | 39.397 | 1364 | 94.8 | 1570 | b, f |
| 1-(2-furanyl)-propan-1-one | 39.582 | 1362 | 73.6 | 1563 |  |
| Nona-3,5-dien-2-one | 39.88 | 1359 | 72.7 |  |  |
| 2-methyl-benzofuran | 40.382 | 1353 | 71.8 | 1576 |  |
| (1-methylethenyl)-pyrazine | 40.755 | 1348 | 74.2 | 1543 |  |
| 2-Acetylpyridine | 40.961 | 1345 | 75.2 | 1597 | f |
| 2-Furanmethanol, propanoate | 41.26 | 1341 | 76.2 | 1602 |  |
| 2-Formyl-1-methylpyrrole | 41.928 | 1330 | 91.9 | 1626 | b, d |
| Acetylpyrazine | 42.367 | 1322 | 75.1 | 1632 | d, f |
| 2,5-dihydro-3,5-dimethyl-2-furanone, | 43.492 | 1299 | 68.9 |  |  |
| 4-(Furan-2-yl)-butan-2-one | 43.643 | 1296 | 83.3 | 1636 |  |
| 2-Furanmethanol | 45.03 | 1261 | 88.1 | 1661 | b, f |
| 3-methylbutanoic acid * | 45.532 | 1247 | 74.8 | 1666 | b, d, f |
| 2-Furfuryl-5-methylfuran | 45.779 | 1240 | 74.3 | 1688 | a, e |
| 2-Acetyl-3-methylpyrazine | 46.177 | 1227 | 81.3 | 1630 | f |
| 2-methyl-3-(2-propenyl)-pyrazin | 47.439 | 1185 | 70.9 |  |  |
| N-acetyl-4(H)-pyridine | 47.609 | 1178 | 71.7 |  | b |
| 2(5H)-Furanone | 49.003 | 1123 | 74.5 | 1743 | e |
| 1-pentyl-pyrrole | 51.729 | 994 | 75 |  |  |
| 1-(2-furanylmethyl)-pyrrole | 53.515 | 893 | 91.9 |  |  |
| 2-methoxy-phenol * | 55.141 | 790 | 88.4 | 1860 |  |
| 2,4-dimethyl-hepta-2,4-dienal | 55.337 | 777 | 75.9 |  |  |
| 3-ethyl-2-hydroxy-2-Cyclopenten-1-one | 56.731 | 680 | 72.5 | 1894 |  |
| 1-(1H-pyrrol-2-yl)-ethanone, | 58.735 | 527 |  | 1973 |  |
| Furfuryl ether | 59.074 | 500 | 73 | 1986 | d |
| Phenol * | 59.494 | 466 | 70.5 | 2000 | b, d, f |
| Formylpyrrole | 59.793 | 441 | 83.3 |  | d |
| 4-ethyl-2-methoxy-phenol * | 59.981 | 425 | 91.6 | 2032 | b, d |
| Furaneol * | 60.144 | 411 | 77 | 2031 |  |
| gamma-Undecalactone | 60.439 | 386 | 70.4 | 2259 | d |
| 1-methylformylpyrrole | 61.188 | 321 | 82 |  |  |
| 4-ethyl-phenol | 62.257 | 225 | 76 | 2187 |  |
| 2-Methoxy-4-vinylphenol * | 62.534 | 200 |  | 2188 | b, d, f |
| 3-hydroxypyridine | 65.054 | -41 | 68.9 |  |  |

^1^ a = Abdelwareth et al. 2021 ; b = Caporaso et al. 2018 ; c = Dippong et al. 2022 ; d = Gonzalez-Rios et al. 2007b ; e = Lee et al. 2017b ; f = Piccino et al. 2014 ; g = Rusinek et al. 2022

**Table S4.** Nucleotide sequence of terpene synthase 10-like (*Ca*TPS10-like), limonene synthase (*Ca*TPS1) and *beta*-Myrcene/Limonene synthase (*Sl*TPS7) for transient expression in *N. benthamiana* leaves. *Ca*TPS1 was characterized by Del Terra et al. (2013) in *C. arabica* drupes and *Sl*TPS7 was characterized by Zhou & Pichersky (2020) in *Solanum lycopersicum* fruits. Genes DNA fragments were synthesized de novo by GenScript® (GenScript, HK Limited, Hong Kong) and cloned into the pBIN61 binary expression vector, under the control of the constitutive CaMV 35S promoter and terminator to generate pBIN61:*Ca*TPS10-like, pBIN61:*Ca*TPS1 and pBIN61:*Sl*TPS7.

| *Ca*TPS10-like | > Coffee terpene synthase 10-like LOC113729710  ATGGCGATCATCAACTTGCCGGTTCCCACCAATTCTTCCAGCGAAGTGAATAAACATAACCATCTCAGGTCCTGTCTCCCTTCCGGGCGAGCTACGTTCACTACTCTTAGTGCTGCAGCCATGAGAAGTGCAACTATGGCAGCGGCTAACGTCCGAGAGCAAAGTGGTCAGAAGCAGCAGCTCATTAATAGACGCTCAGGGAACTACGAAGCTCCACTCTGGGAATTCGATTACATTCAGTCATTGAAAAATGAATATGCGGGTGATATTTACGTCAGTCGGGCTAATGAGTTGAAGGAGCAAGTGAAGATGATGCTCGACGAGGAAGATATGAAGCTGCTGGATTGCATGGAGCTTGTTGACGGGTTGGAAAGGCTAGGACTGGCTTATCACTTTGAGGGTCGAATCAACAGACTATTAAGCAGCGATTACAAAGCTATTCATGAAGGCAATCATCAAAGAAACAAAGAGGATTTGTATGCTGCTGCTCTCGAATTTAGAATCTTCAGGCAAAATGGCTTTAACGTCCCTCAAGATATATTCAATGATTTCATAACTGAGGATGGTGAATTTGATGAAAGCCTTTCTGAGGATACAATGGGACTGCTAAGTTTGTATGAAGCATCTTTCCTGTCGTTGGAAGGTGAAGCCACCCTGGATTTGGCAAGGGAATTCACAACTAAGCACCTCAATAATTATCTAGGCAAGGAAAATACTGATCAAAATCTCAGGATTTTAGTGTACCATGCACTAGAGCTTCCCCTGAGGTGGAGAGCGCCGAGGATAGAAGCTAGGTGGTACATCGATGCATACGAGAGAAGTCCCAACGTGAATCCTACTCTACTTGAGCTTGCAAAAATAGACTTCAACATTGTTCAAGCAATACATCAGCAGGACCTAAAACATGTGTCCTGGTGGTGGAAGAACATACGAATCGCGGAAAAGTTGACATTTATCAGGGACAGGATAGTGGAGAATTTCTTTTGGGCAATAGGAGCTGTCTTCGAGCCTCAGTACGGAAGTTGTCGAAGAATGCTCACCAAGGTCTTTGCTTTGATTACAATGATAGATGACATATACGATGTTTATGGAACTTTGGAAGAATTGGAACTTTTTACTGATGCAGTTGACAGGTGGGATGTCAAAGCCATAGATCAACTTCCAGACTACATGAGAGTTGGATATCTTGGATTTTTCAATTCCATCAACGAGATGGCCTATGACGCTCTCAAAGAGCAAGGCGTACATATAGTGGAATACCTAAGAAAAGTGTGGGCAGATCTGTGTAAAGCATACTTACAAGAGGCAAAATGGTACTACGCTGGATACACACCAACAGTGGAGGAATACCTGGAAAATGCATGGGTTTCAATGTCGGTTCCGGTAATGTTAATGCATGCTTATGCAGGGGTTACCAATCCCATGAATAAGGAAGCCATGGATGTCCTAGACACCCACGATATCGTTCGCTGCTCTTCATATCTTCTACGATTTGCAGATGATTTAGGAACATCACCAGGGGAGATGAAAAGAGGTGATGTCCCGAAATTGGTGCAATGTTACATGAAGGAAGCAGGTTGTTCAGAAGAAGAGTCGAGGGAACATGTATGGTTTTTGCTGAGGGAGACGTGGAAGAAGATGAACAAGGACAGTGAATGGGCGGAATCGCCTTTTTCCAAGACTTTTGTTACAGCTGCAAAGAACTTTGGAAGAGTGGCCCTGGTGATGTACCAATACGGAGATGGGCATGGCCTTCATTCCAATCCTGAGGCTAAGGATCGCATCTTGGCATCACTCTTCTCCCCAGTCCCGCCTGCGTAG |
| --- | --- |
| *Ca*TPS1 | > CCM43927.1 coffee limonene synthase  ATGAGAAGTGCAACTATGGCAGCGGCTAACGTCCGAGAGCAAAGTGGTCAGAAGCAGCAGCTCATTAATAGACGCTCAGGGAACTACGAAGCTCCACTCTGGGAATTCGATTACATTCAGTCATTGAAAAATGAATATGCGGGTGATATTTACGTCAGTCGGGCTAATGAGTTGAAGGAGCAAGTGAAGATGATGCTCGACGAGGAAGATATGAAGCTGCTGGATTGCATGGAGCTTGTTGACGGGTTGGAAAGGCTAGGACTGGCTTATCACTTTGAGGGTCGAATCAACAGACTATTAAGCAGCGATTACAAAGCTATTCATGAAGGCAATCATCAAAGAAACAAAGAGGATTTGTATGCTGCTGCTCTCGAATTTAGAATCTTCAGGCAAAATGGCTTTAACGTCCCTCAAGATATATTCAATGATTTCATAACTGAGGATGGTGAATTTGATGAAAGCCTTTCTGAGGATACAATGGGACTGCTAAGTTTGTATGAAGCATCTTTCCTGTCGTTGGAAGGTGAAGCCACCCTGGATTTGGCAAGGGAATTCACAACTAAGCACCTCAATAATTATCTAGGCAAGGAAAATACTGATCAAAATCTCAGGATTTTAGTGTACCATGCACTAGAGCTTCCCCTGAGGTGGAGAGCGCCGAGGATAGAAGCTAGGTGGTACATCGATGCATACGAGAGAAGTCCCAACGTGAATCCTACTCTACTTGAGCTTGCAAAAATAGACTTCAACATTGTTCAAGCAATACATCAGCAGGACCTAAAACATGTGTCCTGGTGGTGGAAGAACATACGAATCGCGGAAAAGTTGACATTTATCAGGGACAGGATAGTGGAGAATTTCTTTTGGGCAATAGGAGCTGTCTTCGAGCCTCAGTACGGAAGTTGTCGAAGAATGCTCACCAAGGTCTTTGCTTTGATTACAATGATAGATGACATATACGATGTTTATGGAACTTTGGAAGAATTGGAACTTTTTACTGATGCAGTTGACAGGTGGGATGTCAAAGCCATAGATCAACTTCCAGACTACATGAGAGTTGGATATCTTGGATTTTTCAATTCCATCAACGAGATGGCCTATGACGCTCTCAAAGAGCAAGGCGTACATATAGTGGAATACCTAAAAAAAGTGTGGGCAGATCTGTGTAAAGCATACTTACAAGAGGCAAAATGGTACTACGCTGGATACACACCAACAGTGGAGGAATACCTGGAAAATGCATGGGTTTCAATGTCGGTTCCGGTAATGTTAATGCATGCTTATGCAGGGGTTACCAATCCCATGAATAAGGAAGCCATGGATGTCCTAGACACCCACGATATCGTTCGCTGCTCTTCATATCTTCTACGATTTGCAGATGATTTAGGAACATCACCAGGGGAGATGAAAAGAGGTGATGTCCCGAAATTGGTGCAATGTTACATGAAGGAAGCAGGTTGTTCAGAAGAAGAGTCGAGGGAACATGTATGGTTTTTGCTGAGGGAGACGTGGAAGAAGATGAACAAGGACAGTGAATGGGCGGAATCGCCTTTTTCCAAGACTTTTGTTACAGCTGCAAAGAACTTTGGAAGAGTGGCCCTGGTGATGTACCAATACGGAGATGGGCATGGCCTTCATTCCAATCCTGAGGCTAAGGATCGCATCTTGGCATCACTCTTCTCCCCAGTCCCGCCTGCGTAG |
| *Sl*TPS7 | > AEM05856.1 tomato *beta*-myrcene/limonene synthase  ATGGTTTCAATATTCAGTAACGCAGGGATGATGATGGTCACCTTCAATAGACCATCATTTACTTGTTTTTCCTCACTCCATCACTACTCTATATCTGCTCGAGGCGCCATCAACAACATTAGTACTCCTATTTCTGCCACAAGGCGTTCGGGGAATTACAAGCCTACCATGTGGGATTTTCAATTTATTCAATCCCTACACAATCCTTATGAGGGAGACAAGTATATGAAGCGTTTAAACGAACTAAAGAAAGAAGTGAAGAAGATGATGATGACGGTGGAGGGATCACATGATGAAGAGTTAGAGAAGTTGGAGTTGATTGATAATTTAGAGAGGCTCGGAGTGAGTTACCACTTTAAAGATGAAATTATGCAAATATTGAGGAGCATTAATATTAATATTAATATAGCCCCACCAGATTCATTATATACCACATCTTTGAAATTTAGACTCTTGAGACAACATGGTTTTCATATCTCACAAGATGTATTGAAAGATTTCAAAGACGAGAATGGAAATCTGAAGCAGAGTATTTGTAAAGACACAAAAGGTATGTTAGAATTATATGAAGCATCATTTCTCTCTACAGAAACTGAAAACACGCTGAAAAGTGCAACAAGATTCACGATGTCACATCTAAAGAATTATGTCGACAATCATTCATGTGGAAATCAAGACGATGATATAATAGTGGAATTAGTGGTCCATGCTTTGGAACTTCCAAGACATTGGATGATGCCAAAATTAGAGACAGAGTGGTATATTAGAATTTATGGGAGAATGCCAAATGCTAATCCTCTTCTGCTGGAGCTTGCAAAGTTGGACTTCAACATTGTCCAAGCAGCACACCAACAAGATTTGAAAATTTTGTCGAGGTGGTGGAAGAGCATGAGTTTGGCAGAGAAGTTATCATTTTCAAGAGATAGACTGGTGGAAGACTTTTTCTGGTCAGTGGGATTAGCATTTGAGCCTCAACACAGCTTGTGTCGAAGAATGTTGGCGAAAAACGTTGCTTTTATAATCGTCATAGATGACATTTATGATGTCTATGGTAGTCTTGATGAGTTGGAAATCTTCACTCATGCTGTTGAAAGATGGGATATAAAAGCAATGGAGCAGCTTCCAGACTACATGAAAATATGTTACCTTTCGCTCTTCAACACTACCAATGAAATGGCCTATCATATTCTCAAACAACAAGGGATTAATGTCCTGCCCTACCTCACAAAACAATGGACAGATTTATGCAAATCATACTTGCAAGAAGCAAAATGGTACCACAATGGGCATAAGCCAAGACTAGAAGAGTACATGGATAATGCATGGATTTCAATTGCAACTCCTTTGGTATTACTCCATGCATTCATCTTTCTCACCAATCCAATAACCCAAGAGGCATTGGAATCCTTGAACAATTATCCAGACATAATTCGTCGGTGTGCTATAATTAATCGTTTCGTCGATGATTTGGGGACATCATCGGATGAATTGAAAAGAGGTGATGTTCCCAAGTCGATACAATGTTACATGAATGACACGGGTGCTTCAGAAGAAGAGGCAAGAGAACACATCAATTTATTGATAAAGGAGATGTGGGAAGTGATGAACAAAGACCAAATTAGTAAACAAGTGCTATTTTCGGAAGAATTCATTAAGATTGTGTTTAATTTTTCAAGAACATCACACTGCGTGTATCAGCATGGAGATGGGCATGGAATTCAAAATTCTCACATAACAAATCGGATTTCCAAATTACTCTTTGAGCCTCTCATTATATAA |

**Table S5.** Green beans volatile compounds mean concentration (μg/g of dry mass) per genotypes (3 biological replicates). One-way analysis of variance of the effects of Genotype on green beans volatile compounds (μg/g of dry mass) of field-grown *Coffea arabica* genotypes at harvest (red stage) (“n.s.” indicates p > 0.05 ; “*” indicates 0.05 > p > 0.01 ; “**” indicates 0.01 > p > 0.001 ; “***” indicates 0.001 > p). Means with different letters are significantly different (Tukey’s HSD test, P < 0.01). The chirality of enantiomeric substances was not determined so that substances such as limonene are either (+)-limonene or (-)-limonene.

| Volatile compounds | ET47 | Geisha  Especial | Marsellesa | T5175 | Significance level |
| --- | --- | --- | --- | --- | --- |
| Dimethyl sulfide | 0.31±0.21 | 0.72±0.01 | 0.5±0.16 | 0.15±0.05 | n.s |
| 2,4-dimethylheptane | 2.69±3.55 | 1.48±1.45 | 0.77±0.23 | 0.88±0.42 | n.s |
| Methyl acetate | 5.83±0.9 | 7.79±0.08 | 5.51±1.61 | 7.18±1.7 | n.s |
| Butanal | 0.32±0.09 | 1.24±0.04 | 0.56±0.16 | 0.94±0.78 | n.s |
| 2-methylbutanal | 0.05±0.01 | 0.12±0.01 | 0.08±0.04 | 0.13±0.06 | n.s |
| 3-methylbutanal | 0.07±0.01 | 0.18±0.05 | 0.12±0.05 | 0.23±0.16 | n.s |
| Ethanol | 9.51±3.52 | 1.56±0.31 | 8.68±4.83 | 6.21±5.35 | n.s |
| Methyl 2-methylbutanoate | 0.57±0.1 | 0.73±0.26 | 0.27±0.05 | 0.65±0.26 | n.s |
| Methyl 3-methylbutanoate | 4.26±1.46 ab | 12.73±5.04 a | 2.65±0.58 b | 3.58±0.76 b | ** |
| 1-Cyclohexyl-2-phenylethane | 1.59±0.23 | 1.38±0.17 | 1.22±0.3 | 1.17±0.32 | n.s |
| 3-*p*-Menthene | 0.24±0.08 b | 0.7±0.09 a | 0.05±0.07 b | 0.01±0 b | *** |
| Ethyl 3-methylbutanoate | 0.98±0.14 | 0.63±0.4 | 0.49±0.06 | 0.42±0.14 | n.s |
| Butyl acetate | 0.81±0.39 | 1.23±0.42 | 0.99±0.27 | 1.69±0.98 | n.s |
| Hexanal | 0.54±0.4 | 1.28±0.25 | 0.5±0.2 | 0.82±0.79 | n.s |
| *beta*-Pinene | 0.17±0.03 b | 0.35±0.04 a | 0.04±0.05 c | 0.01±0 c | *** |
| *p*-Menth-1-ene | 0.58±0.1 b | 1.86±0.28 a | 0.12±0.14 bc | 0.03±0.03 c | *** |
| Methyl 3-methylpentanoate | 0.19±0.03 ab | 0.58±0.24 a | 0.16±0.07 b | 0.2±0.01 ab | ** |
| Limonene | 2.49±0.98 ab | 4.48±0.43 a | 0.36±0.31 bc | 0.17±0.19 c | *** |
| 3-methylbutan-1-ol | 0.89±0.21 | 0.51±0.02 | 0.86±0.08 | 0.61±0.12 | n.s |
| 2-pentylfuran | 0.55±0.25 | 0.22±0.07 | 0.3±0.26 | 0.27±0.11 | n.s |
| Butyl 3-methylbutanoate | 0.36±0.13 | 0.82±0.62 | 0.15±0.11 | 0.23±0.05 | n.s |
| *p*-Cymene | 0.69±0.08 b | 1.89±0.46 a | 0.13±0.16 b | 0.05±0.03 b | *** |
| Terpinolene | 0.02±0.02 b | 0.12±0 a | 0.01±0.01 b | 0±0 b | *** |
| 1-methylheptyl acetate | 0.62±0.24 | 0.31±0.02 | 0.59±0.28 | 0.38±0.13 | n.s |
| Hexan-1-ol | 2.41±0.87 | 2.11±0.03 | 2.07±1.21 | 1.85±1.81 | n.s |
| Octan-3-ol | 0.2±0.09 | 0.23±0 | 0.14±0.08 | 0.15±0.06 | n.s |
| 2-methoxy-3-(2-methylpropyl)-pyrazine | 0.14±0.02 | 0.39±0.04 | 0.35±0.38 | 0.85±0.21 | n.s |
| 2-phenylethanal | 0.11±0.1 | 0.21±0.04 | 0.18±0.21 | 0.18±0.11 | n.s |
| 3-methylbutanoic acid | 1.68±1.48 b | 7.48±2.54 a | 0±0 b | 0.02±0.02 b | ** |
| Methyl 2-phenylacetate | 0.23±0.12 | 0.1±0.03 | 0.15±0.12 | 0.27±0.19 | n.s |
| 2-phenylethanol | 0.9±0.42 | 0.45±0.03 | 0.72±0.37 | 0.52±0.18 | n.s |

**Table S6.** Roasted beans volatile compounds mean concentration (μg/g of dry mass) per genotypes (3 biological replicates). One-way analysis of variance of the effects of Genotype on roasted beans volatile compounds (μg/g of dry mass) of field-grown *C. arabica* genotypes at harvest (red stage) and 1300m elevation. Means with different letters are significantly different (Tukey’s HSD test, P < 0.01). The chirality of enantiomeric substances was not determined so that substances such as limonene are either (+)-limonene or (-)-limonene.

| Volatile compounds | ET47 | Geisha Especial | Marsellesa | T5175 | Significance level |
| --- | --- | --- | --- | --- | --- |
| Acetone | 96.69±11.79 | 98.93±6.47 | 90.55±26.1 | 95.06±6.87 | n.s |
| Methyl acetate | 34.81±16.68 | 44.43±1.49 | 29.21±10.66 | 24.77±2.85 | n.s |
| 2-methylfuran | 40.83±12.12 | 41.28±2.02 | 35.83±12.39 | 34.49±3.67 | n.s |
| butan-2-one | 28.19±5.17 | 24.51±4.4 | 25.22±5.52 | 25.53±2.84 | n.s |
| 2-methylbutanal | 21.91±2.08 | 21.83±3.29 | 25.56±8.72 | 23.83±2.01 | n.s |
| 3-methylbutanal | 13.61±1.29 | 11.08±2.14 | 13.41±3.46 | 13.45±1.62 | n.s |
| Ethanol | 4.95±1.36 | 4.6±1.63 | 4.95±0.81 | 4.26±1.45 | n.s |
| 2,5-dimethylfuran | 4.75±1.06 | 4.35±0.43 | 3.94±1.47 | 3.55±0.18 | n.s |
| Butan-2,3-dione | 44.2±6.02 | 35.6±0.56 | 40.26±8.94 | 38.59±6.68 | n.s |
| Pentan-2,3-dione | 40.14±10.1 | 27.74±1.2 | 35.22±2.56 | 31.02±6.23 | n.s |
| 2-Vinylfuran | 3.7±0.27 | 3.2±0.96 | 2.99±0.92 | 2.94±0.31 | n.s |
| Pent-3-en-2-one | 2.41±0.4 | 2.2±0.09 | 2.06±0.81 | 2.37±0.14 | n.s |
| Hexan-2,3-dione | 4.92±0.65 | 4.15±0.52 | 4.31±0.57 | 4.14±0.43 | n.s |
| 1-methyl-1H-pyrrole | 8.44±1.95 | 8.39±0.74 | 6.06±3.71 | 8±0.3 | n.s |
| Hexan-3,4-dione | 4.7±0.72 | 4.03±0.12 | 4.34±0.19 | 4.11±0.57 | n.s |
| *beta*-Myrcene | 0.86±0.07 | 1.5±0.08 | 1.23±0.58 | 0.63±0.05 | n.s |
| Pyridine | 274.09±72.72 | 325.74±15.18 | 207.11±92.84 | 257.51±11.5 | n.s |
| Limonene | 2.11±0.24 b | 6.3±0.57 a | 1.26±1.02 b | 0.77±0.2 b | *** |
| Pyrazine | 27.39±4.52 | 27.69±0.69 | 27.39±8.62 | 31.86±3.52 | n.s |
| 2-methyl-pyridine | 2.58±0.62 | 2.17±0.25 | 2.43±0.79 | 2.49±0.25 | n.s |
| Furan, 2-(methoxymethyl)- | 3.98±0.87 | 5.33±1.08 | 4.14±0.73 | 2.96±0.26 | n.s |
| *alpha*-Ocimene | 0.82±0.11 | 1.29±0.03 | 1.06±0.41 | 0.54±0.1 | n.s |
| dihydro-2-methyl-3(2H)-furanone | 50.61±7.92 | 48.83±6.19 | 47.61±3.47 | 38.52±4.39 | n.s |
| methyl-pyrazine | 235.01±31.42 | 259.28±4.83 | 251.36±57.44 | 297.23±44.5 | n.s |
| 3-hydroxy-butan-2-one | 18.16±2.39 | 19.29±2.03 | 20.22±1.4 | 18.15±2.61 | n.s |
| 1-hydroxy-2-propanone | 124.69±29.02 | 121.64±0.66 | 132.44±3.27 | 114.95±13.78 | n.s |
| 2,5-dimethyl-pyridine | 108.48±16.77 | 123.13±4.8 | 117.46±33.3 | 143.26±18.19 | n.s |
| 2,6-dimethyl-pyridine | 116.99±16.65 | 132.19±4.69 | 125.71±35.12 | 157.21±19.26 | n.s |
| Ethyl-pyrazine | 47.65±4.89 | 50.56±0.36 | 52.55±12.82 | 65.98±8.04 | n.s |
| 2,3-dimethyl-pyrazine | 21.35±2.71 | 23.69±1.14 | 22.77±6.36 | 26.82±3.17 | n.s |
| 2-Hydroxy-3-pentanone | 6.61±1.32 | 6.54±1.13 | 6.97±0.58 | 5.87±0.7 | n.s |
| 3-methyl-2-cyclopenten-1-one | 3.44±0.53 | 3.52±0.1 | 3.17±1.11 | 2.81±0.25 | n.s |
| 1-Hydroxy-2-butanone | 15.08±4.5 | 14.8±0.74 | 16.35±1.32 | 13.66±0.99 | n.s |
| 3-ethyl-pyridine | 5.94±2.08 | 6.51±0.9 | 5.1±3.04 | 5.77±0.62 | n.s |
| 2-Ethyl-6-methylpyrazine | 60.48±10.3 | 71.25±0.42 | 68.37±19.98 | 95.81±8.29 | n.s |
| 2-Ethyl-5-methylpyrazine | 34.7±6.14 | 37.63±0.99 | 38.86±11.87 | 50.53±3.95 | n.s |
| 2-Ethyl-3-methylpyrazine | 45.03±7.36 | 55.23±0.29 | 50.51±15.99 | 67.04±6.32 | n.s |
| 2-propylpyrazine | 2.87±0.62 | 3.07±0.17 | 3.39±1.32 | 4.38±0.08 | n.s |
| 1-Hydroxy-2-pentanone | 127.97±53.11 | 129.11±0.77 | 161.82±24.51 | 113.82±7.89 | n.s |
| Acetic acid | 159±35.76 | 166.8±25.24 | 170.81±15.45 | 139.53±11.45 | n.s |
| 2,5-dimethyl-3-ethylpyrazine | 259.89±61.23 | 268.1±21.71 | 295.82±28.9 | 238.49±16.08 | n.s |
| Furfural | 261.8±65.21 | 237.89±64.15 | 272.55±32.04 | 225.44±24.84 | n.s |
| 1-(acetyloxy)-2-propanone | 286.18±31.26 | 277.73±29.63 | 314.12±70.83 | 272.56±33.22 | n.s |
| Linalool oxide | 2±0.09 b | 4.03±0.53 a | 2.72±0.41 ab | 1.76±0.37 b | ** |
| Furfuryl methyl sulfide | 7.77±2.97 | 6.72±0.65 | 7.06±2.34 | 7.05±1.3 | n.s |
| 2-ethenyl-6-methyl-pyrazine | 7.67±1.19 | 8.15±0.13 | 7.79±2.91 | 9.32±0.59 | n.s |
| Furfuryl formate | 21.46±1.6 | 21.61±1.86 | 23.43±6.23 | 22.06±2.63 | n.s |
| Acetylfuran | 126.22±3.37 | 133.64±4.77 | 124.8±15.2 | 99.7±4.68 | n.s |
| Benzaldehyde | 13.77±4.44 | 11.48±0.58 | 11.03±5.88 | 13.95±2.71 | n.s |
| 2-Butylfuran | 13.74±0.75 | 14.22±0.48 | 14.3±2.24 | 12.78±1.07 | n.s |
| 2-methoxy-3-(2-methylpropyl)-pyrazine | 0.51±0.1 b | 1.52±0.13 ab | 1.02±0.53 b | 2.9±0.43 a | *** |
| 2-Furanmethanol, acetate | 144.01±19.11 | 157.97±5.39 | 143.18±38.81 | 125.09±18.4 | n.s |
| Linalool | 0.81±0.21 | 1.46±0.34 | 1.28±0.63 | 0.6±0.1 | n.s |
| 5-Methylfurfural | 295.27±60.14 | 278.85±53.6 | 313.75±34.39 | 253.68±24.36 | n.s |
| 1-(2-furanyl)-propan-1-one | 16.93±0.98 | 17.11±0.04 | 16.3±2.51 | 13.71±0.64 | n.s |
| Nona-3,5-dien-2-one | 10.17±2.29 | 11.58±1.27 | 10.95±4.22 | 8.99±0.48 | n.s |
| 2-methyl-benzofuran | 2.42±0.48 | 2.75±0.3 | 2.15±0.75 | 1.7±0.2 | n.s |
| (1-methylethenyl)-pyrazine | 4.92±0.74 | 5.37±0.11 | 6.12±2.42 | 8.12±0.72 | n.s |
| 2-Acetylpyridine | 2.35±0.39 | 2.49±0.09 | 2.44±0.83 | 2.55±0.15 | n.s |
| 2-Furanmethanol, propanoate | 10.4±1.55 | 10.18±0.96 | 11.59±2.66 | 8.95±1.55 | n.s |
| 2-Formyl-1-methylpyrrole | 39.63±2.88 | 47.04±2.95 | 37.84±10.35 | 42.14±1.76 | n.s |
| Acetylpyrazine | 2.27±0.44 | 2.82±0.88 | 3.03±0.69 | 4.07±0.87 | n.s |
| 2,5-dihydro-3,5-dimethyl-2-furanone, | 5.25±0.49 | 4.93±0.12 | 5.24±2.04 | 4.01±0.66 | n.s |
| 4-(Furan-2-yl)-butan-2-one | 4.8±0.6 | 5.03±0.09 | 4.8±1.53 | 4.12±0.47 | n.s |
| 2-Furanmethanol | 470.58±39.89 | 466.92±35.63 | 473.84±153.71 | 488.02±55.42 | n.s |
| 3-methylbutanoic acid | 44.08±7.79 b | 95.43±14.14 a | 27.59±3.04 b | 23.34±4.1 b | *** |
| 2-Furfuryl-5-methylfuran | 10.6±3.06 | 11.34±0.77 | 9.79±4.46 | 8.29±1.52 | n.s |
| 2-Acetyl-3-methylpyrazine | 10.49±1.67 | 11.37±1.16 | 11.82±3.63 | 15.72±1.61 | n.s |
| 2-methyl-3-(2-propenyl)-pyrazine | 3.11±0.94 | 3.28±0.19 | 3.54±1.71 | 4.37±0.22 | n.s |
| N-acetyl-4(H)-pyridine | 20.81±1.79 | 19.07±1.25 | 21.26±7.68 | 19.7±1.52 | n.s |
| 2(5H)-Furanone | 13.62±1.11 | 12.05±0.88 | 14.99±3.48 | 13.69±1.3 | n.s |
| 1-pentyl-pyrrole | 9.91±0.48 | 9.15±0.28 | 10.43±3.64 | 9.83±0.73 | n.s |
| 1-(2-furanylmethyl)-pyrrole | 36.94±3.09 | 35.67±1.7 | 39.37±15.11 | 40.97±2.1 | n.s |
| 2-methoxy-phenol | 39.11±17.07 | 17.88±0.82 | 15.12±10.43 | 17.32±2.13 | n.s |
| 2,4-dimethyl-hepta-2,4-dienal | 23.83±1.93 a | 22.23±2.98 ab | 22.04±2.02 ab | 15.12±1.34 b | ** |
| 3-ethyl-2-hydroxy-2-Cyclopenten-1-one | 7.43±0.27 | 7.08±0.66 | 8.8±3.65 | 8.23±0.68 | n.s |
| 1-(1H-pyrrol-2-yl)-ethanone, | 39.5±4.18 | 37.81±1.28 | 43.06±15.6 | 44.17±2.77 | n.s |
| Furfuryl ether | 15.07±3.94 | 16.44±1.2 | 16.26±7.57 | 14.72±3.33 | n.s |
| Phenol | 16.25±5.85 | 13.88±0.45 | 13.58±7.35 | 10.82±0.46 | n.s |
| Formylpyrrole | 45.24±5.59 | 42.78±4.18 | 51.55±7.64 | 49.69±3.52 | n.s |
| 4-ethyl-2-methoxy-phenol | 24.59±5.67 | 33.73±5.05 | 28.42±15.11 | 39.86±2.05 | n.s |
| Furaneol | 8.66±1.64 | 7.27±0.16 | 10.47±1.58 | 8.41±1.81 | n.s |
| gamma-Undecalactone | 6.11±0.45 | 5.51±1.1 | 6.03±0.98 | 4.23±0.14 | n.s |
| 1-methylformylpyrrole | 10.07±0.96 | 9.54±0.46 | 11.67±3.11 | 11.73±0.74 | n.s |
| 4-ethyl-phenol | 1.81±0.36 | 2.19±0.31 | 1.76±0.83 | 1.58±0.15 | n.s |
| 2-Methoxy-4-vinylphenol | 29.59±2 | 33.93±3.37 | 36.03±16.47 | 55.47±11.42 | n.s |
| 3-hydroxypyridine | 10.87±0.7 | 10.77±0.35 | 12.44±3.27 | 12.07±1.87 | n.s |

**Table S7.** Description of odors and flavors associated with Geisha Especial discriminating free monoterpenes. Descriptions are given according to THE GOOD SCENTS COMPANY. Crosses (X) indicate that no description is available.

| FREE Monoterpenes | odor/flavor type ASSOCIATED WITH Monoterpenes |
| --- | --- |
| Limonene | Citrus/Citrus |
| *beta*-Pinene | Herbal/Pine |
| *p*-Cymene | Terpenic/Terpenic |
| Terpinolene | Herbal/Woody |
| 3-*p*-Menthene | X |
| *p*-Menth-1-ene | X |
| Linalool oxide | Floral/Green |
| *beta*-Myrcene | Spicy/Woody |
| Linalool | Floral/Citrus |

**Table S8.** Isoprenoid biosynthetic genes among the 2609 DEGs that displayed a higher expression level in the Geisha Especial genotype. Biosynthetic segment, Arabica genes ID, Arabidopsis thaliana orthologous genes and Arabica genes putative functions. Mean level of expression (normalized counts) of the isoprenoid biosynthetic genes, for each genotype and ripening stage. Means were calculated for the three biological replicates. P-value for the genotype effect: one-way analysis of variance of the effects of Genotype on the level of expression (normalized counts) of field-grown *C. arabica* genotypes at both stages.

| Biosynthetic segment | Arabica genes ID (J.Hopkins genome) | Arabidopsis thaliana orthologous genes | Putative function | | | ET47_Red | | ET47_Yellow | | Geisha Especial_Red | | Geisha Especial_Yellow | | Marsellesa_Red | | Marsellesa_Yellow | | T5175_Red | | T5175_Yellow | | P.value (genotype effect) |
| --- | --- | --- | --- | --- | --- | --- | --- | --- | --- | --- | --- | --- | --- | --- | --- | --- | --- | --- | --- | --- | --- | --- |
| Prenyl-pyrophosphate precursor synthesis | LOC113701753 | AT1G76490 | 3-hydroxy-3-methylglutaryl-coenzyme A reductase 1-like | | | 28.34 | | 45.13 | | 31.79 | | 82.17 | | 12.03 | | 25.29 | | 16.32 | | 35.89 | | 0.0141 |
|  | LOC113704431 | AT2G26930 | 4-diphosphocytidyl-2-C-methyl-D-erythritol kinase, chloroplastic/chromoplastic-like | | | 2.24 | | 2.57 | | 3.05 | | 3.65 | | 1.51 | | 1.83 | | 1.51 | | 2.37 | | 0.000822 |
|  | LOC113708033 | AT5G60600 | 4-hydroxy-3-methylbut-2-en-1-yl diphosphate synthase (ferredoxin), chloroplastic-like | | | 14.89 | | 17.86 | | 40.97 | | 36.39 | | 13.49 | | 21.99 | | 7.66 | | 14.04 | | 0.00000388 |
|  | LOC113742146 | AT1G74470 | geranylgeranyl diphosphate reductase, chloroplastic | | | 1.15 | | 2.55 | | 1.70 | | 4.10 | | 0.44 | | 1.61 | | 0.67 | | 0.89 | | 0.00616 |
|  | LOC113687778 | AT4G36810 | heterodimeric geranylgeranyl pyrophosphate synthase small subunit, chloroplastic-like | | | 0.24 | | 0.69 | | 0.65 | | 2.82 | | 0.09 | | 0.53 | | 0.17 | | 0.82 | | 0.0523 |
|  | LOC113690509 | AT4G36810 | heterodimeric geranylgeranyl pyrophosphate synthase small subunit, chloroplastic-like | | | 0.28 | | 0.50 | | 0.65 | | 2.42 | | 0.27 | | 0.69 | | 0.09 | | 0.61 | | 0.0187 |
|  | LOC113708766 | AT3G02780 | isopentenyl-diphosphate Delta-isomerase I | | | 14.50 | | 20.63 | | 21.37 | | 29.70 | | 15.00 | | 24.38 | | 12.11 | | 27.39 | | 0.308 |
|  | LOC113709314 | AT3G02780 | isopentenyl-diphosphate Delta-isomerase I-like | | | 48.25 | | 69.29 | | 50.78 | | 74.56 | | 29.96 | | 55.84 | | 30.95 | | 74.09 | | 0.439 |
|  | LOC113727754 | AT5G60600 | LOW QUALITY PROTEIN: 4-hydroxy-3-methylbut-2-en-1-yl diphosphate synthase (ferredoxin), chloroplastic-like | | | 8.35 | | 9.72 | | 16.15 | | 19.44 | | 3.40 | | 6.31 | | 3.96 | | 7.50 | | 0.000000378 |
|  | LOC113692757 | AT4G15560 | probable 1-deoxy-D-xylulose-5-phosphate synthase 2, chloroplastic | | | 13.72 | | 30.04 | | 26.99 | | 55.75 | | 3.36 | | 14.14 | | 5.47 | | 18.44 | | 0.00268 |
|  | LOC113695854 | AT4G15560 | probable 1-deoxy-D-xylulose-5-phosphate synthase 2, chloroplastic | | | 3.71 | | 8.18 | | 9.93 | | 23.12 | | 1.21 | | 3.94 | | 1.25 | | 3.39 | | 0.000247 |
| Monoterpenoid | LOC113730856 | AT1G61680 | (3S,6E)-nerolidol synthase 1-like | | | 0.80 | | 0.36 | | 1.33 | | 1.91 | | 0.29 | | 0.89 | | 0.43 | | 0.38 | | 0.0157 |
|  | LOC113729710 | AT3G25830 | terpene synthase 10-like | | | 9.10 | | 26.65 | | 14.22 | | 51.24 | | 0.43 | | 5.12 | | 0.03 | | 0.66 | | 0.00124 |
|  | LOC113712611 | AT4G24220 | iridoid synthase CYC2-like | | | 10.61 | | 21.75 | | 14.57 | | 28.94 | | 6.08 | | 11.67 | | 5.41 | | 9.13 | | 0.0106 |
| Sesquiterpenoid | LOC113723870 | AT4G16730 | alpha-farnesene synthase-like | | | 3.35 | | 13.75 | | 5.73 | | 14.88 | | 0.73 | | 4.48 | | 0.18 | | 1.09 | | 0.0126 |
|  | LOC113723866 | AT4G16730 | alpha-farnesene synthase-like | | | 0.56 | | 1.20 | | 0.96 | | 1.57 | | 0.08 | | 0.48 | | 0.05 | | 0.49 | | 0.014 |
| Diterpenoid | LOC113702909 | AT1G79460 | cis-abienol synthase, chloroplastic-like isoform X1 | | | 2.46 | | 4.17 | | 2.57 | | 4.94 | | 0.47 | | 2.33 | | 1.13 | | 2.82 | | 0.0428 |
|  | LOC113714615 | AT5G25900 | ent-kaurene oxidase, chloroplastic-like | | | 11.98 | | 10.91 | | 12.07 | | 11.67 | | 7.54 | | 6.32 | | 6.94 | | 5.65 | | 0.00000159 |
|  | LOC113725096 | AT5G51810 | gibberellin 20 oxidase 1-D-like isoform X1 | | | 0.38 | | 0.42 | | 1.83 | | 1.15 | | 0.31 | | 0.40 | | 0.27 | | 0.34 | | 0.00199 |
| Triterpenoid | LOC113731220 | AT4G34640 | squalene synthase-like | | | 6.50 | | 12.13 | | 10.49 | | 15.44 | | 2.41 | | 6.84 | | 3.22 | | 7.05 | | 0.00151 |
| Monoterpene indole alkaloid | LOC113708505 | AT1G22360 | 7-deoxyloganetic acid glucosyltransferase-like | | | 12.04 | | 18.31 | | 16.59 | | 19.01 | | 15.13 | | 17.69 | | 6.68 | | 8.94 | | 0.00148 |
|  | LOC113726633 | AT1G22400 | 7-deoxyloganetin glucosyltransferase-like | | | 17.09 | | 12.17 | | 49.42 | | 23.73 | | 18.86 | | 16.99 | | 8.12 | | 8.38 | | 0.0000222 |
|  | LOC113694042 | AT3G57030 | protein STRICTOSIDINE SYNTHASE-LIKE 10-like | | | 1.60 | | 1.37 | | 2.88 | | 2.34 | | 1.50 | | 1.46 | | 0.83 | | 0.97 | | 0.00182 |
|  | LOC113697149 | AT3G57030 | protein STRICTOSIDINE SYNTHASE-LIKE 10-like | | | 0.41 | | 0.44 | | 0.58 | | 1.32 | | 0.25 | | 0.45 | | 0.26 | | 0.35 | | 0.0149 |
|  | LOC113711450 | AT3G26040 | vinorine synthase-like | | | 1.33 | | 3.30 | | 2.03 | | 3.99 | | 0.33 | | 1.48 | | 0.31 | | 0.78 | | 0.00926 |
|  | LOC113714113 | AT3G26040 | vinorine synthase-like | | | 0.41 | | 0.94 | | 0.60 | | 2.30 | | 0.19 | | 0.79 | | 0.24 | | 0.70 | | 0.14 |
| Modification of the carbon backbone | LOC113725249 | AT2G24190 | | (+)-neomenthol dehydrogenase-like isoform X1 | 18.99 | | 17.01 | | 28.62 | | 24.68 | | 14.30 | | 10.80 | | 21.45 | | 20.05 | | 0.0000055 | |
|  | LOC113706877 | AT2G26170 | | cytochrome P450 711A1-like isoform X1 | 0.20 | | 0.10 | | 0.54 | | 0.27 | | 0.06 | | 0.17 | | 0.07 | | 0.02 | | 0.00754 | |
|  | LOC113732030 | AT5G24910 | cytochrome P450 714C2-like | | | 15.97 | | 41.38 | | 33.28 | | 71.67 | | 6.09 | | 19.48 | | 8.61 | | 19.70 | | 0.00107 |
|  | LOC113718378 | AT4G31500 | cytochrome P450 71A1-like | | | 3.31 | | 8.48 | | 5.92 | | 14.46 | | 2.78 | | 10.34 | | 4.23 | | 13.40 | | 0.467 |
|  | LOC113738743 | AT3G48270 | cytochrome P450 71A8-like | | | 1.99 | | 1.85 | | 3.93 | | 3.05 | | 1.92 | | 2.35 | | 0.80 | | 0.83 | | 0.000353 |
|  | LOC113711378 | AT5G36220 | cytochrome P450 81D1-like | | | 0.79 | | 0.90 | | 1.42 | | 0.98 | | 0.23 | | 0.60 | | 0.37 | | 0.58 | | 0.0121 |
|  | LOC113699283 | AT4G37370 | cytochrome P450 81E8-like | | | 0.97 | | 1.44 | | 2.09 | | 2.89 | | 0.82 | | 1.41 | | 0.84 | | 1.08 | | 0.0162 |
|  | LOC113702162 | AT4G37370 | cytochrome P450 81E8-like | | | 6.82 | | 12.90 | | 11.01 | | 24.28 | | 2.40 | | 5.88 | | 2.27 | | 4.86 | | 0.00153 |
|  | LOC113692279 | AT4G31500 | cytochrome P450 83B1-like | | | 0.43 | | 0.59 | | 1.96 | | 3.59 | | 0.25 | | 0.26 | | 0.11 | | 0.02 | | 0.00000248 |
|  | LOC113698998 | AT3G50660 | cytochrome P450 90B1 | | | 3.24 | | 8.06 | | 8.66 | | 19.24 | | 0.88 | | 3.57 | | 0.92 | | 4.28 | | 0.000523 |
|  | LOC113692035 | AT2G27690 | cytochrome P450 94C1-like | | | 0.16 | | 0.36 | | 0.36 | | 0.73 | | 0.05 | | 0.17 | | 0.07 | | 0.21 | | 0.00228 |
|  | LOC113695406 | AT2G27690 | cytochrome P450 94C1-like | | | 0.13 | | 0.27 | | 0.36 | | 0.76 | | 0.06 | | 0.30 | | 0.09 | | 0.09 | | 0.00685 |
|  | LOC113743378 | AT4G23160 | cytochrome P450 97B2, chloroplastic-like | | | 66.45 | | 39.79 | | 94.46 | | 69.24 | | 72.81 | | 45.36 | | 77.99 | | 40.93 | | 0.11 |
|  | LOC113690604 | AT3G14640 | cytochrome P450 CYP72A219-like | | | 39.10 | | 38.29 | | 77.71 | | 69.06 | | 32.10 | | 36.52 | | 41.96 | | 40.48 | | 0.0000115 |
|  | LOC113698228 | AT4G36220 | cytochrome P450 CYP736A12-like | | | 3.22 | | 2.48 | | 11.94 | | 6.76 | | 4.10 | | 5.05 | | 2.00 | | 2.88 | | 0.000117 |
|  | LOC113698521 | AT4G36220 | cytochrome P450 CYP736A12-like | | | 1.66 | | 1.55 | | 6.93 | | 7.24 | | 3.54 | | 3.62 | | 3.57 | | 3.44 | | 0.00000286 |
|  | LOC113701514 | AT4G36220 | cytochrome P450 CYP736A12-like | | | 0.39 | | 0.10 | | 1.62 | | 0.57 | | 0.17 | | 0.56 | | 0.25 | | 0.31 | | 0.00879 |
|  | LOC113712365 | AT4G31940 | cytochrome P450 CYP82D47-like | | | 0.76 | | 1.14 | | 0.98 | | 1.53 | | 0.37 | | 0.59 | | 0.55 | | 0.88 | | 0.00717 |

**Fig. S1.** (A) Experimental set up: Four genotypes with three trees of the same genotype (biological replicates) were grown at 1300 m in barrels under a transparent shelter to control inputs and flowering initiation and to reduce environmental variability. (B) Berry collected at the ‘yellow stage’. (C) Berry collected at the ‘red stage’, showing the endosperm.


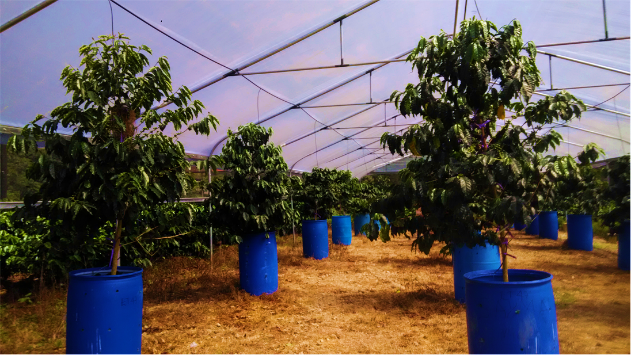

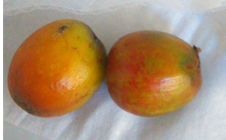

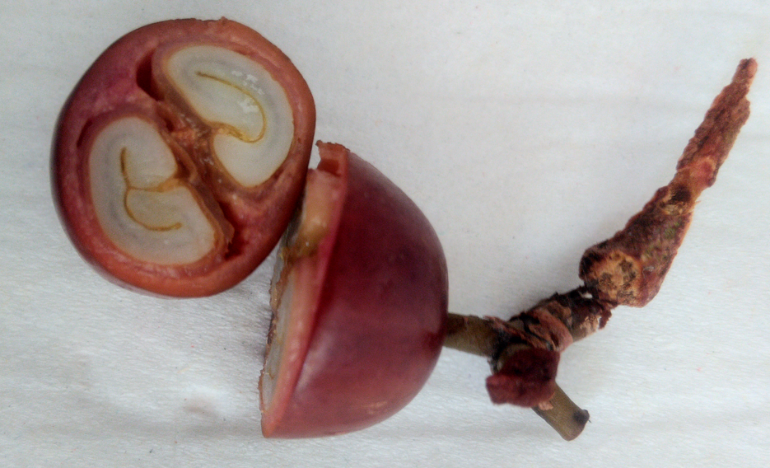


**A**

**B**

**C**


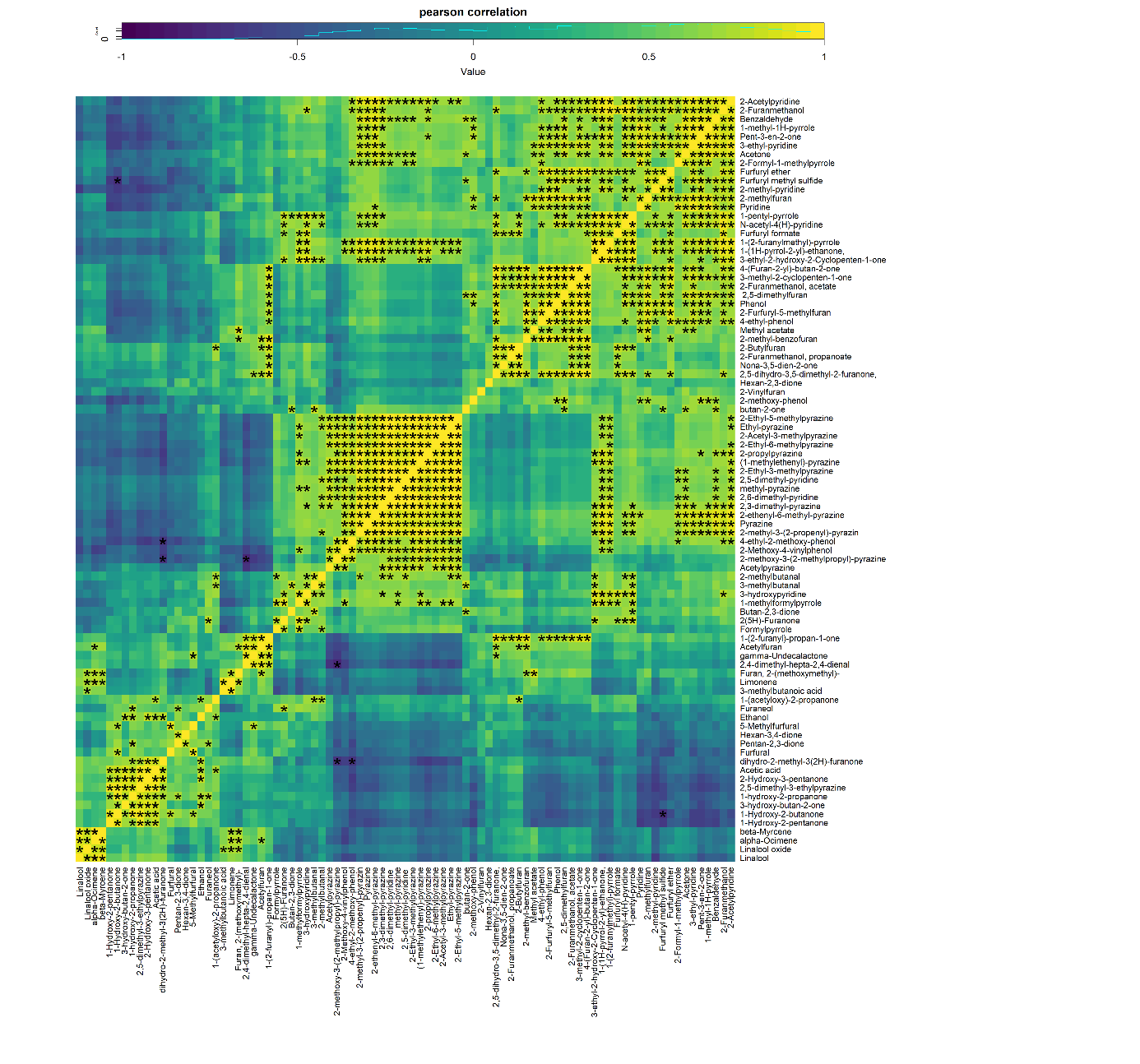


**Fig. S2.** Heatmap of content correlations (Pearson’s rank correlation from -1 to +1) of roasted beans volatile compounds. Cells marked with a black star indicate significant correlation with an r ≥ 0.7 for positive correlation and r ≤ -0.7 for negative correlation and p < 0.05.


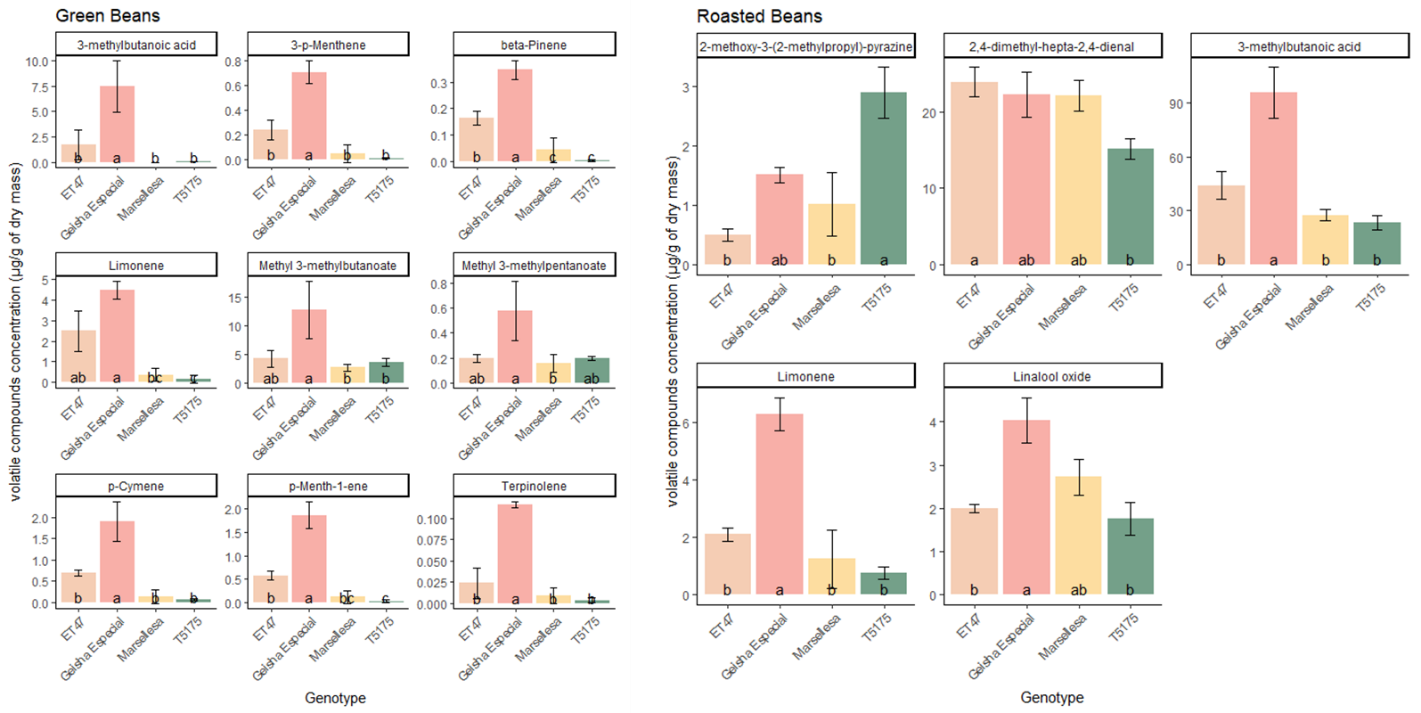

**Fig. S3.** Green and roasted beans volatile compounds mean concentration (μg/g of dry mass) per genotypes (3 biological replicates). Means were calculated for the three biological replicates and are expressed in µg/g of dry mass. Means with different letters are significantly different (Tukey’s HSD test, P < 0.01). Error bars indicate standard deviation (SD) obtained from three biological replicates. The chirality of enantiomeric substances was not determined so that substances such as limonene are either (+)-limonene or (-)-limonene.


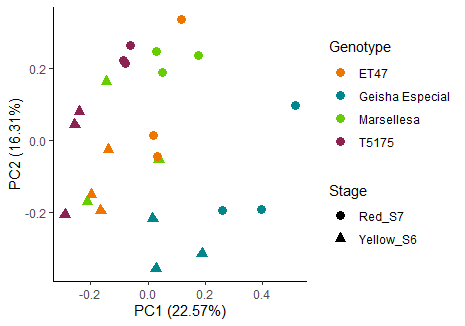


**Fig. S4.** Principal component analysis (PCA) of the gene expression data (normalized read counts) of the 24 samples (two fruit ripening stages x four genotypes x three replicates).


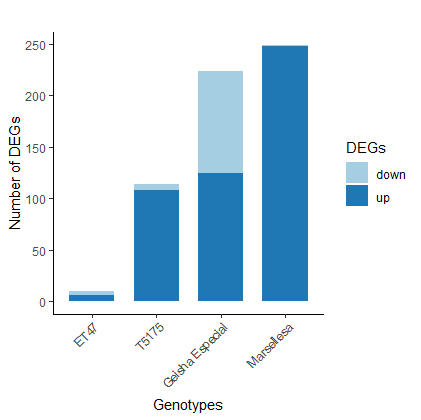


**Fig. S5.** Number of differentially expressed genes (DEGs) between the ‘yellow stage’ and the ‘red stage’ for each of the four genotypes. Cut-off of LogFC ≥ 1 for up-regulated genes or LogFC ≤ -1 for down-regulated genes, and FDR < 0.01.


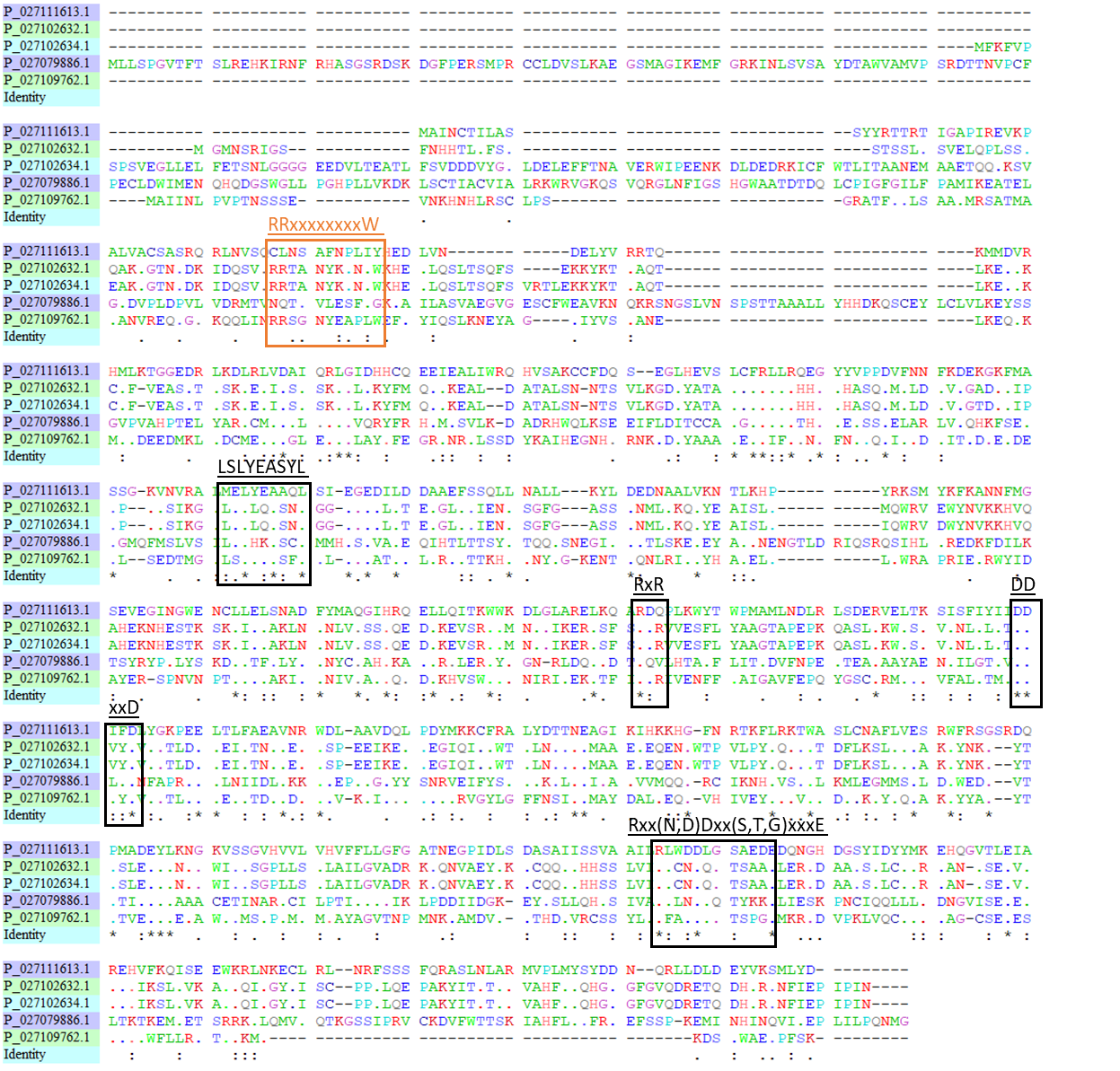


**Fig. S6.** Protein sequence alignment of the five *Coffea arabica* terpene synthases. The protein sequences of (3S,6E)-nerolidol synthase 1-like (P_027111613.1), alpha-farnesene synthase-like (P_027102632.1 and P_027102634.1), cis-abienol synthase, chloroplastic-like isoform X1 (P_027079886.1) and terpene synthase 10-like (P_027109762.1) were aligned. Those five TPS were up-regulated in Geisha Especial beans. Conserved amino acid regions typical of terpene synthases are in black boxes (« RxR », « DDxxD », « Rxx(N,D)Dxx(S,T,G)xxxE », « LSLYEASYL »). Conserved amino acid regions typical of terpene synthases involved in cyclization of monoterpenes are in orange boxes (« RRxxxxxxxxW »).


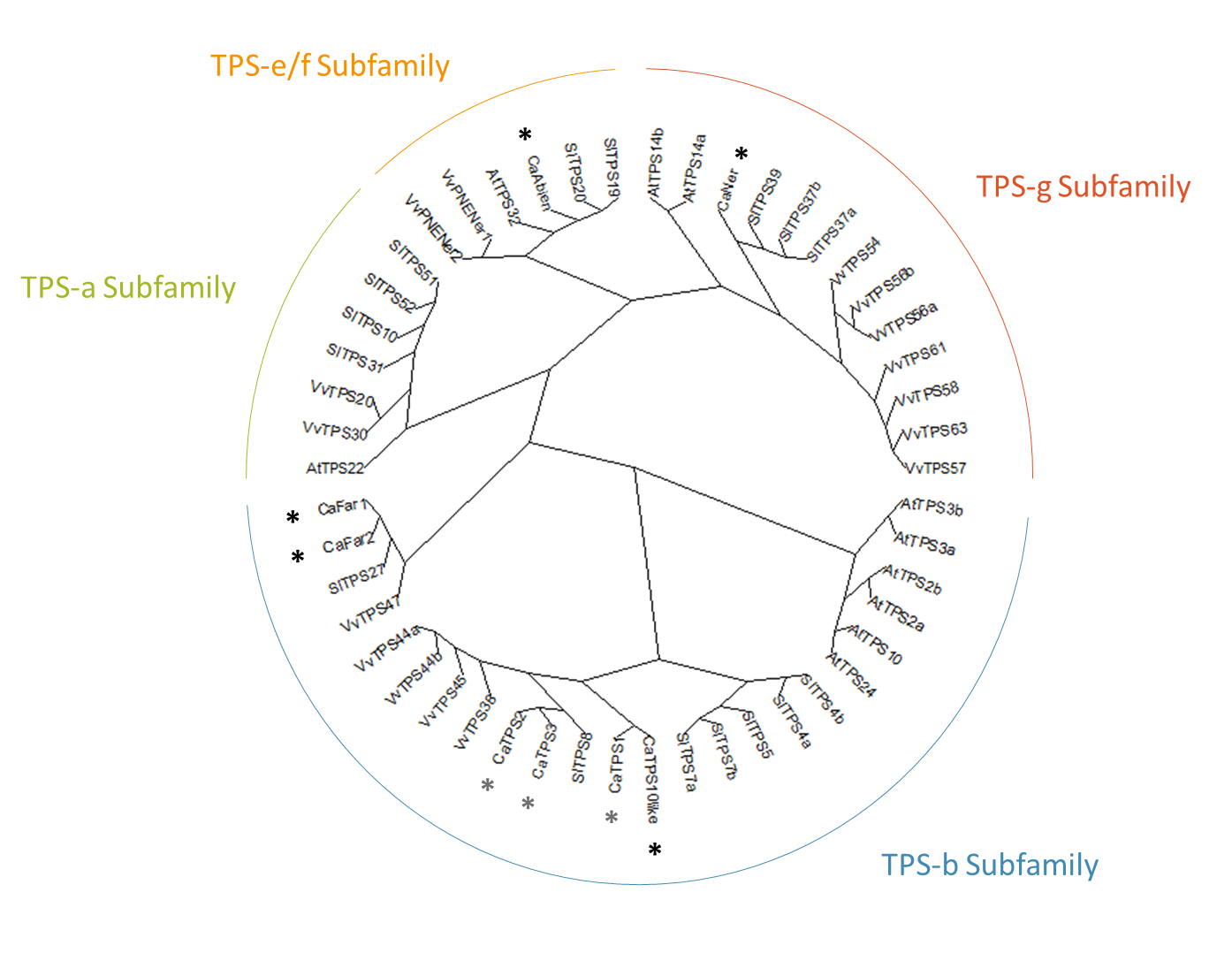


**Fig. S7.** Phylogenetic tree analysis of terpene protein sequences from *Arabidopsis thaliana* (At), *Vitis vinifera* (Vv), *Solanum lycopersicum* (Sl) and *Coffea arabica* (Ca), constructed by Neighbor-joining (NJ) method. The amino acid sequences were obtained from the National Center for Biotechnology Information database and were analyzed and visualized using the ape R package. The black stars correspond to the protein sequences isolated from the Caturra Red cultivar and the grey stars correspond to the protein sequences isolated from the Catuai Red cultivar in the Del Terra *et al.* (2013) study.


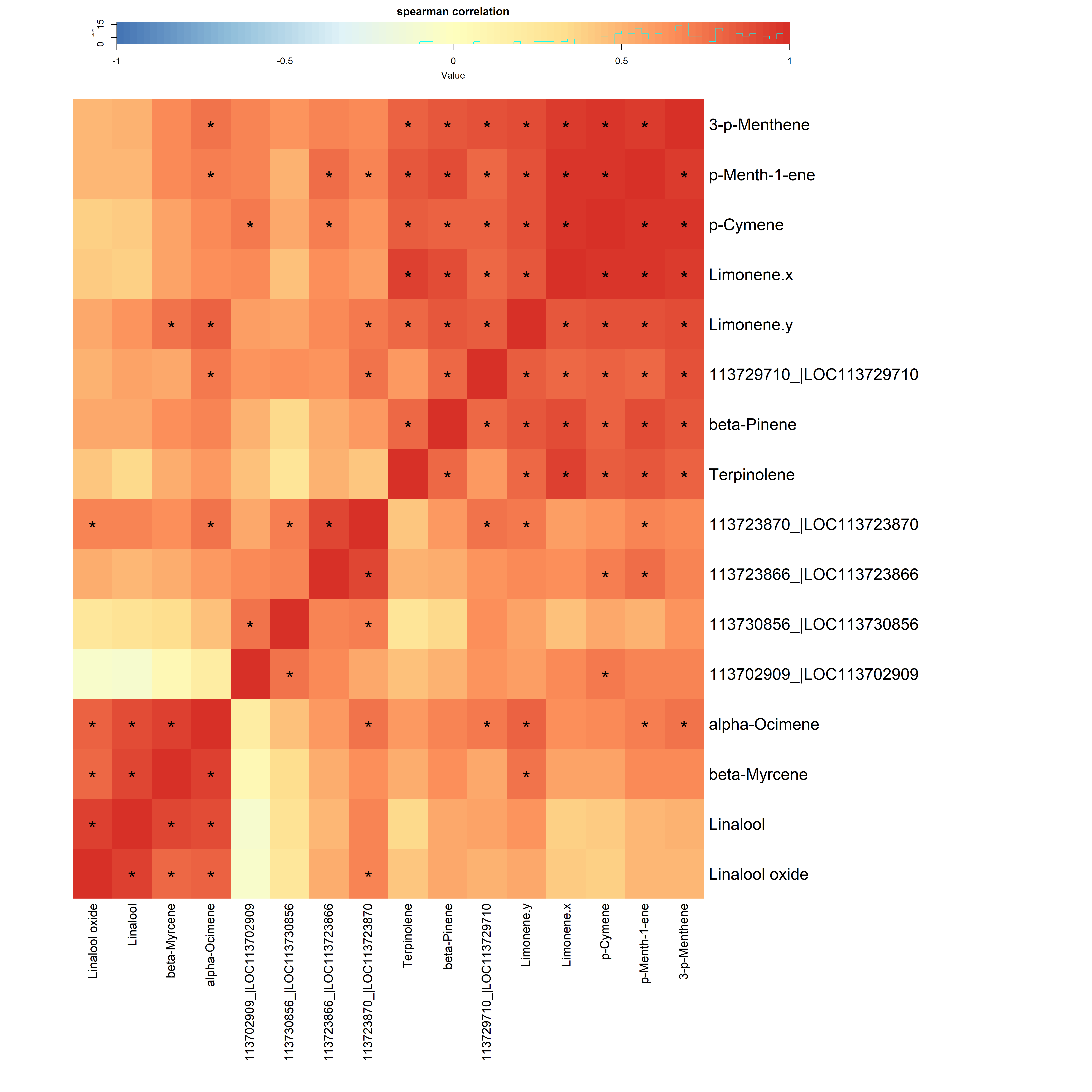


**Fig. S8.** Spearman’s correlation between the expression level of the five terpene synthases up-regulated in Geisha Especial beans and the free monoterpenes found in green and roasted beans (Limonene.x = green beans; limonene.y = roasted beans).


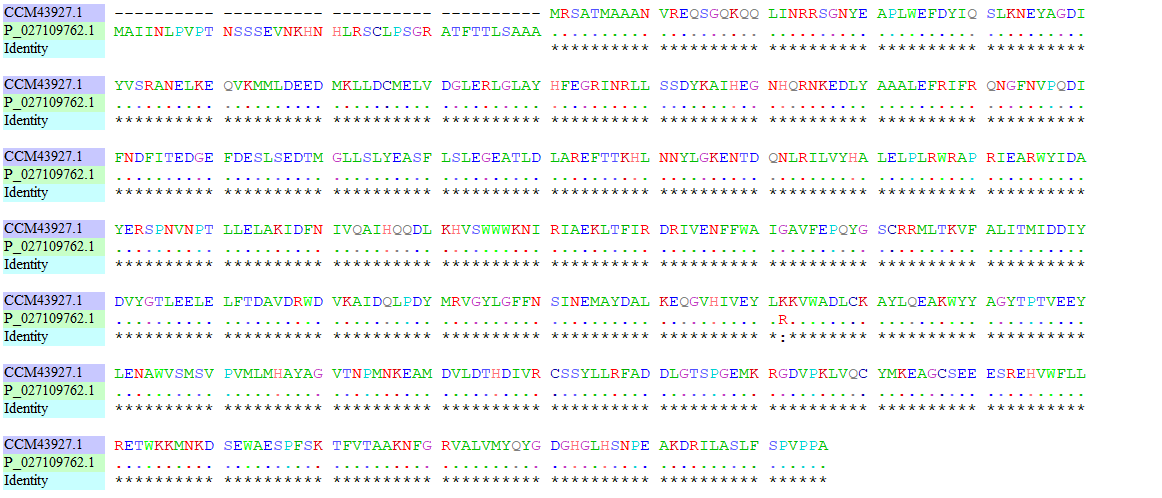


**Fig. S9.** Protein sequence alignement of the two TPS, limonene synthase [Coffea arabica] (CCM43927.1) isolated from cultivar Catuai Red (Del Terra et al., 2013), and terpene synthase 10-like [Coffea arabica] (P_027109762.1), isolated from cultivar Caturra red


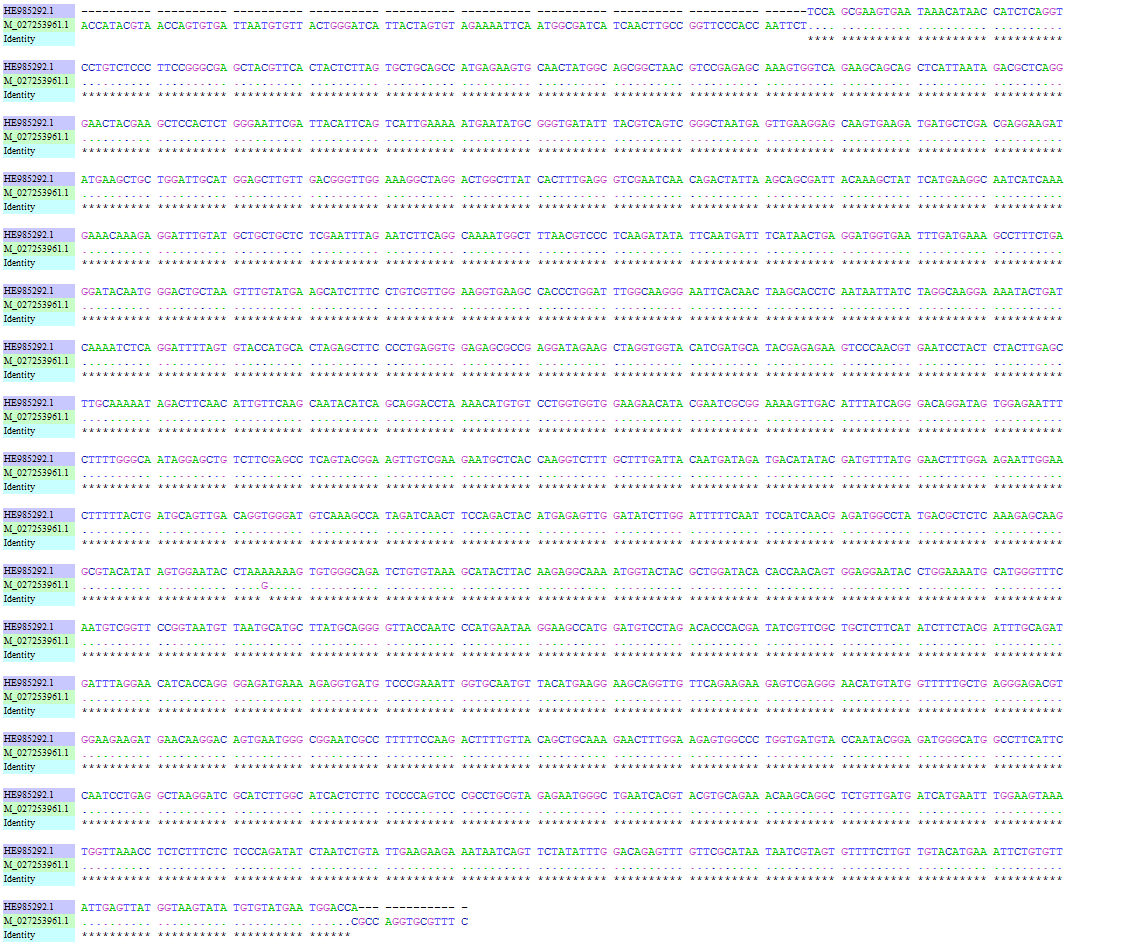


**Fig. S10.** Nucleotide sequence alignment (mRNA) of the two TPS, limonene synthase (TPS1) [Coffea arabica] (HE985292.1) isolated from cultivar Catuai Red (Del Terra et al., 2013), and terpene synthase 10-like [Coffea arabica] (M_027253961.1), isolated from cultivar Caturra red

***beta*-Pinene**


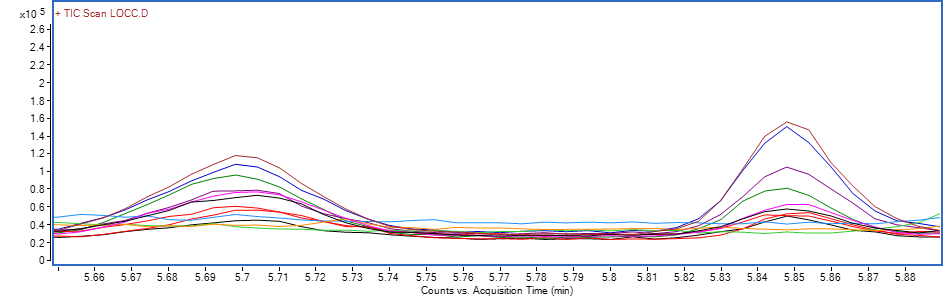


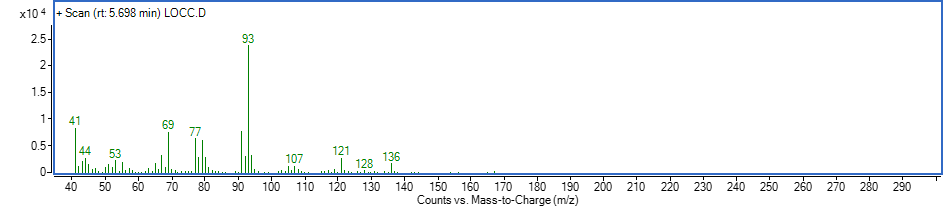


**Pseudolimonen**


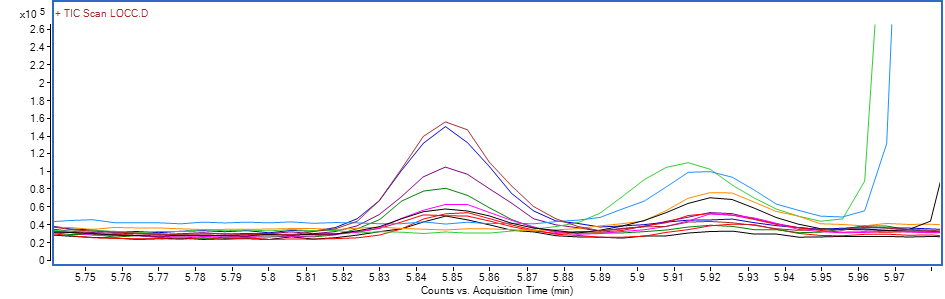


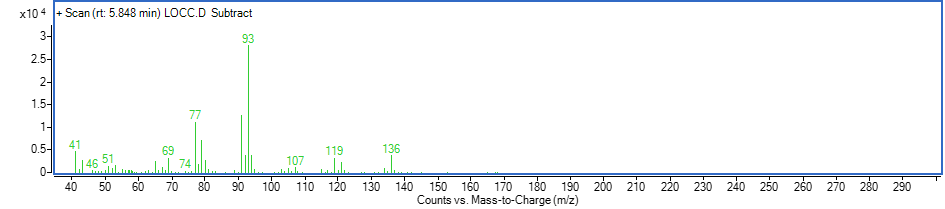


**2-Penten-1-ol, (Z)-**


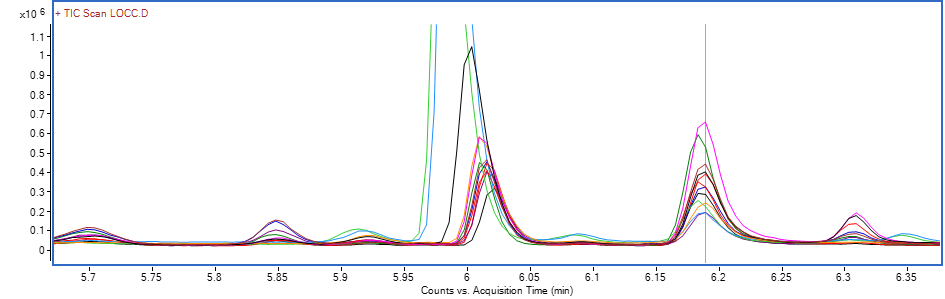


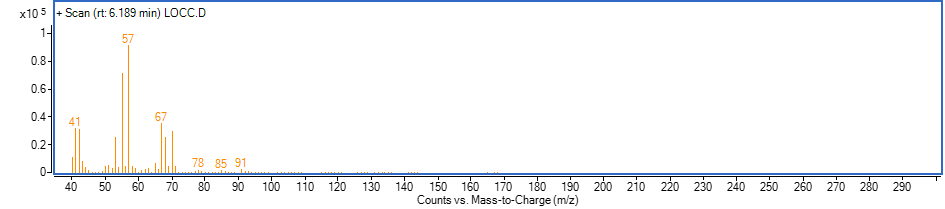


***beta*- Myrcene**


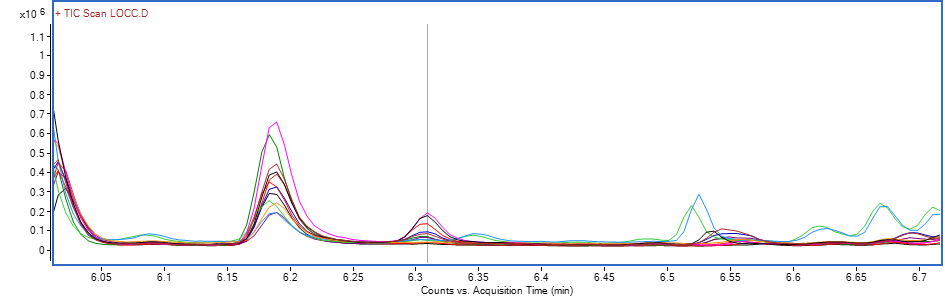


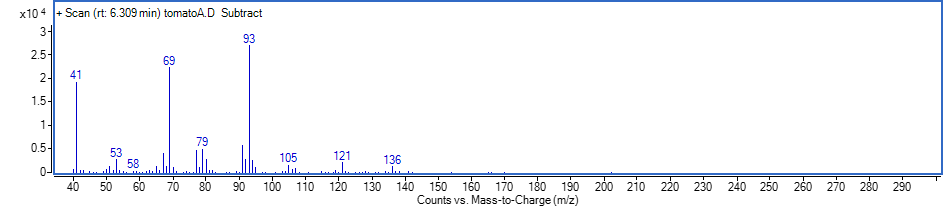


**Limonene**


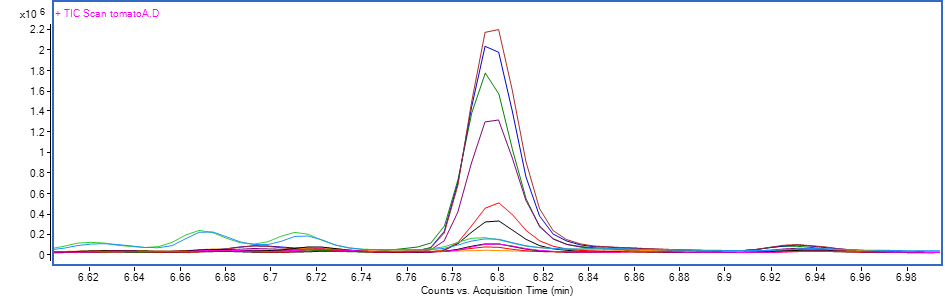


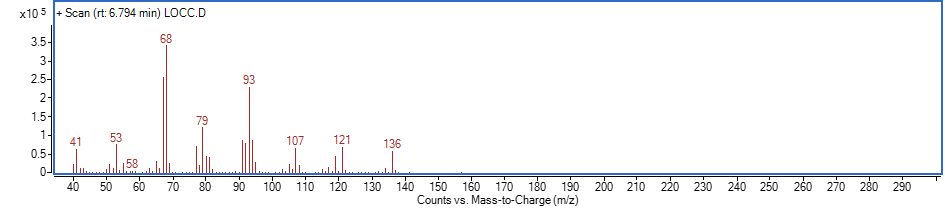


**Eucalyptol**


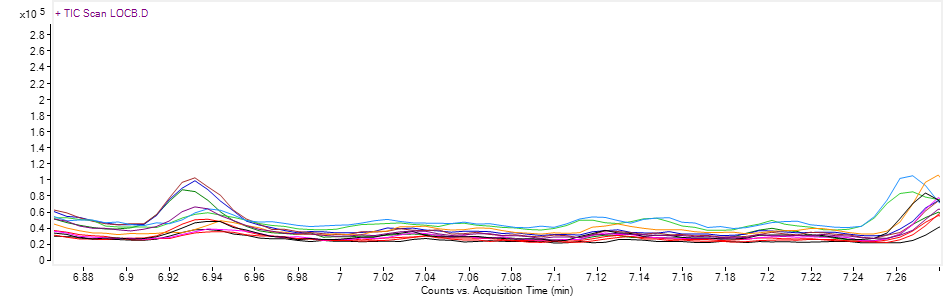


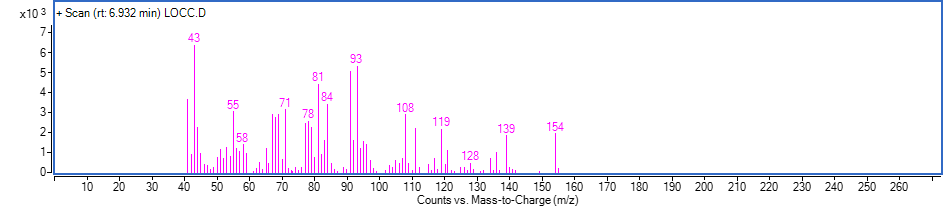


***alpha*-terpinene**


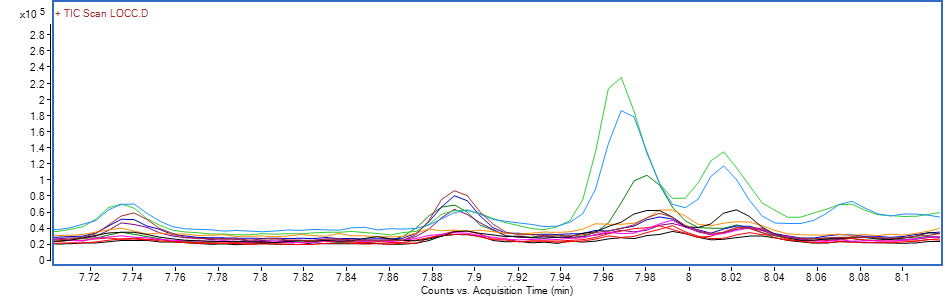


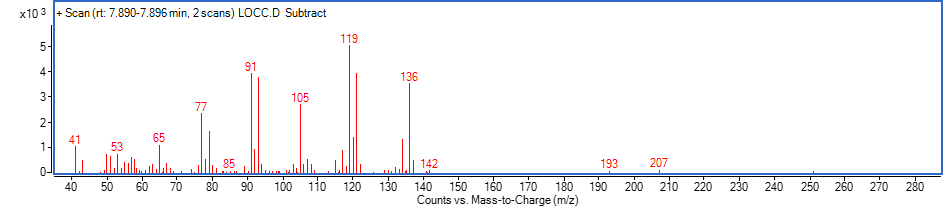


**Fig. S11.** Characterisation of products generated by overexpression of TPS genes using transient gene expression in *N. benthamiana.* GC-MS chromatograms and MS spectrum results of the *N. benthamiana* leaves volatile compounds following transient expression (35S:TPS1 - black, red, dark green lines -, 35:TPS10-like - dark blue, purple, brown lines -, 35S:TPS7 - pink, red, black lines -, and 35S:pBIN61 empty vector - light blue, light green, orange lines -).

**References**

Abdelwareth, A., Zayed, A., & Farag, M. A. (2021). Chemometrics-based aroma profiling for revealing origin, roasting indices, and brewing method in coffee seeds and its commercial blends in the Middle East. *Food Chemistry*, 349, 129-162.

Bertrand, B., Boulanger, R., Dussert, S., Ribeyre, F., Berthiot, L., Descroix, F., & Joët, T. (2012). Climatic factors directly impact the volatile organic compound fingerprint in green Arabica coffee bean as well as coffee beverage quality. *Food Chemistry*, 135, 2575–2583.

Caporaso, N., Whitworth, M. B., Cui, C., & Fisk, I. D. (2018). Variability of single bean coffee volatile compounds of Arabica and robusta roasted coffees analysed by SPME GC-MS. *Food Research International*, 108, 628–640.

Dippong, T., Dan, M., Kovacs, M. H., Kovacs, E. D., Levei, E. A., & Cadar, O. (2022). Analysis of Volatile Compounds, Composition, and Thermal Behavior of Coffee Beans According to Variety and Roasting Intensity. *Foods*, 11, 3146, 1-15. https://doi.org/10.3390/foods11193146

Gonzalez-Rios, O., Suarez-Quiroz, M. L., Boulanger, R., Barel, M., Guyot, B., Guiraud, J. P., & Schorr-Galindo, S. (2007a). Impact of “ecological” post-harvest processing on the volatile fraction of coffee beans. I. Green coffee. *Journal of Food Composition and Analysis*, 20, 289–296.

Gonzalez-Rios, O., Suarez-Quiroz, M. L., Boulanger, R., Barel, M., Guyot, B., Guiraud, J. P., & Schorr-Galindo, S. (2007b). Impact of “ecological” post-harvest processing on coffee aroma: II. Roasted coffee. *Journal of Food Composition and Analysis*, 20, 297–307.

Hadj Salem, F., Lebrun, M., Mestres, C., Sieczkowski, N., Boulanger, R., & Collignan, A. (2020). Transfer kinetics of labeled aroma compounds from liquid media into coffee beans during simulated wet processing conditions. *Food Chemistry*, 322, 1-7. https://doi.org/10.1016/j.foodchem.2020.126779

Holscher, W. and Steinhart, H. (1995). Aroma compounds in green coffee. In Food Flavors. Generation, Analysis and Process Influence, Charalambous G., Ed., Elsevier Science, Amsterdam 37A, 785-803

Lee, K.-G., & Shibamoto, T. (2002). Analysis of volatile components isolated from Hawaiian green coffee beans (Coffea arabica L.). *Food and Fragrance Journal*, 17, 349–351.

Lee, L. W., Tay, G. Y., Cheong, M. W., Curran, P., Yu, B., & Liu, S. Q. (2017a). Modulation of the volatile and non-volatile profiles of coffee fermented with Yarrowia lipolytica: I. Green coffee. LWT – *Food Science and Technology*, 77, 225–232.

Lee, L. W., Tay, G. Y., Cheong, M. W., Curran, P., Yu, B., & Liu, S. Q. (2017b). Modulation of the volatile and non-volatile profiles of coffee fermented with Yarrowia lipolytica: II. Roasted coffee. LWT – *Food Science and Technology*, 80, 32–42.

Piccino, S., Boulanger, R., Descroix, F., & Sing, A. S. C. (2014). Aromatic composition and potent odorants of the “specialty coffee” brew “Bourbon Pointu” correlated to its three trade classifications. *Food Research International*, 61, 264–271.

Pereira, G. V. M., Neto, E., Soccol, V. T., Medeiros, A. B. P., Woiciechowski, A. L., & Soccol, C. R. (2015). Conducting starter culture-controlled fermentations of coffee beans during on-farm wet processing: Growth, metabolic analyses and sensorial effects. *Food Research International*, 75, 348–356.

Rusinek, R., Dobrzanski, B., Jr., Oniszczuk, A., Gawrysiak-Witulska, M., Siger, A., Karami, H., Ptaszynska, A. A., Zytek, A., Kapela, K., & Gancarz, M. (2022). How to Identify Roast Defects in Coffee Beans Based on the Volatile Compound Profile. *Molecules*, 27, 8530, 1-13. https://doi.org/10.3390/molecules27238530

Scheidig, C., Czerny,M., & Schieberle, P. (2007). Changes in Key Odorants of Raw Coffee Beans during Storage under Defined Conditions. *J. Agric. Food Chem.*, 55, 5768-5775

Vezzulli, F., Lambri, M., & Bertuzzi, T. (2023). Volatile Compounds in Green and Roasted Arabica Specialty Coffee: Discrimination of Origins, Post-Harvesting Processes, and Roasting Level. *Foods*, 12, 489. https://doi.org/10.3390/foods12030489

Yeretzian, C., Jordan, A., Badoud, R., & Lindinger, W. (2002). From the green bean to the cup of coffee: investigating coffee roasting by on-line monitoring of volatiles. *European Food Research and Technology*, 214, 92–104. https://doi.org/10.1007/s00217-001-0424-7
